# Microbial response to long-term spatially stratified phosphorus application in Northeast China

**DOI:** 10.3389/fpls.2025.1669876

**Published:** 2025-10-23

**Authors:** Liyuan Hou, Bing Han, Yixin Wang, Xiaoli Wang, Wuliang Shi, Ning Cao, Yubin Zhang

**Affiliations:** College of Plant Science, Jilin University, Changchun, China

**Keywords:** spatially stratified fertilization, phosphorus efficiency, maize yield, microbial community structure, functional genes

## Abstract

As a critical factor influencing crop productivity in agricultural ecosystems, phosphorus (P)-fertilizer application can significantly alter soil physicochemical properties. However, the relative efficiencies of different types of spatially stratified P fertilizers and their underlying biological mechanisms remain insufficiently elucidated. In this study, an 8-year field experiment was conducted in a black soil region of Northeast China to compare the effects of five P-fertilization regimes: CK (without P application), FP (100% as basal fertilizer), APP (20% as starter fertilization by ammonium polyphosphate), MAP (20% as starter fertilization by monoammonium phosphate), and CMP (20% as starter fertilization by calcium magnesium phosphate). We systematically investigated the effects of spatially stratified P fertilization on soil physical properties, nutrient accumulation, maize yield performance, and bacterial and fungal community structure. CMP demonstrated the best performance in improving soil aeration and enhancing water infiltration capacity. MAP significantly increased the soil total P content by 18.62% and the soil Olsen-P content by 81.46% compared to those of FP. Both MAP and CMP promoted P uptake in various parts of maize plants, including the roots, straw, and grains. All tested starter P fertilizers improved P use efficiency. Compared to that of FP, the soil P surplus was reduced by 7.52%, 14.74%, and 13.04% under APP, MAP, and CMP, respectively. MAP demonstrated the most pronounced yield-increasing effect. Based on amplicon sequencing (16S rRNA for bacteria, interspacer region for fungi) and microbiome profiling, this study confirms that fungi are more susceptible than bacteria to variations in fertilizer types and application methods. Furthermore, the relative abundance of *Tausonia* was most significantly influenced by MAP. By enhancing the relative abundance of P-cycling functional genes (*gph, phoU*), MAP modulated the abundance of dominant microbial taxa such as Acidobacteria and Proteobacteria, thereby significantly improving maize yield. Therefore, in maize cropping systems in the black soil region of Northeast China, optimized P fertilizer selection and application methods can effectively reduce soil P surplus and modulate microbial community structure and functional diversity while maintaining stable crop yields.

## 1 Introduction

In intensive agricultural systems, the continuous phosphorus (P)-fertilizer application employed to sustain high productivity leads to excessive soil P accumulation and associated environmental risks (MacDonald et al., 2011; Withers et al., 2014; Zhang et al., 2018; Zhu et al., 2018). Moreover, such practices may severely compromise terrestrial biodiversity and critical ecosystem functions (MacDonald et al., 2011; Withers et al., 2014; Zhang et al., 2018). Furthermore, as a finite and geographically concentrated resource, global phosphate rock reserves are projected to meet fertilizer demands only for several decades to centuries (MacDonald et al., 2011; Withers et al., 2014; Zhang et al., 2018). China exhibits significant potential to improve P use efficiency (PUE). Strategic optimization of P-fertilizer application can substantially reduce input requirements while simultaneously enhancing fertilizer utilization rates and crop productivity (Du et al., 2020). These approaches primarily include integrated organic-inorganic fertilization, fertigation systems, and localized nutrient placement techniques. However, most existing studies have narrowly focused on single-layer soil fertilization, overlooking the comprehensive impacts of fertilization practices on crop nutrient acquisition and utilization efficiency (Hansel et al., 2017; Wu et al., 2021).

Deep fertilization enhances nutrient acquisition efficiency by optimizing the soil nutrient distribution and balancing nutrient availability, thereby promoting crop root absorption of nitrogen (N), P, and potassium (K). This method induces the development of spatially efficient root architectures and establishes a synergistic relationship between soil nutrients and crops, representing a key strategy for improving the cultivated soil layer structure (Sun et al., 2024; Wang et al., 2022). Specifically, stratified fertilization not only stimulates root system development but also significantly enhances both crop yield and quality (Gao et al., 2024). Moreover, starter fertilizer is typically band-applied near the seeds to minimize P fixation through limited soil-fertilizer contact, thereby enhancing P availability for the seedlings during early germination. The efficacy of starter P in promoting the early growth vigor of maize is well-established, particularly in low-P soils (Preston et al., 2019; Vetsch and Randall, 2002). Therefore, systematic investigation of stratified fertilization techniques and P source selection is critical to identify the key contributors to maize yield. Moreover, optimizing P formulations that maximize yield gains under reduced input regimes can help to realize the ultimate goal of achieving both agricultural productivity and soil health sustainability.

Microorganisms constitute fundamental components of soil P-cycling, while playing pivotal roles in modulating plant AP. Previous studies have shown the important roles of soil microorganisms in mediating soil nutrient cycling and plant–soil interactions (Hinsinger et al., 2009). Notably, P-cycling microbial communities serve as key drivers of soil P transformation and P-fertilizer utilization efficiency (Lang et al., 2021). To date, research in this field has predominantly focused on elucidating the effects of P-fertilizer application rates on the soil microbial community structure (Lang et al., 2021; Liu et al., 2024a, b). Long-term P fertilization significantly affects soil fungal and bacterial communities (Beauregard et al., 2010). The abundance of most microbial taxa, including key bacterial and fungal guilds, exhibits dose-dependent responses to P input levels (Huang et al., 2016). Furthermore, soil microorganisms exert a critical regulatory role in driving nutrient mineralization and biogeochemical cycling throughout plant growth (Regehr et al., 2015). Notably, the dynamic fluctuations in rhizosphere microbial abundance and diversity across successive crop developmental stages significantly modulate soil nutrient availability (Su et al., 2024). Moreover, soil microbial communities demonstrate remarkable adaptive capacity, maintaining functional and structural stability across diverse soil conditions under 37 years of differential nutrient input regimes (Hamel et al., 2006). These previous findings demonstrate that the soil microbial community composition is shaped by complex and dynamic interactions among soil environmental factors, fertilization regimes, and crop species.

Certain keystone microbial taxa play a pivotal role in soil P cycling by mediating the transformation of both inorganic phosphorus (Pi) and organic phosphorus (Po) into plant-available forms (Khan et al., 2007; Sharma et al., 2013). The associated genes were classified into four functional categories according to their roles in soil P-cycling (Dai et al., 2020; Hartman et al., 2017; Mahmood et al., 2020), including P-starvation response (PSR) regulation, the P-uptake and transport system, Pi solubilization, and Po mineralization, and exhibited distinct distribution patterns across agricultural ecosystems (Bergkemper et al., 2016; Dai et al., 2020). For instance, genes encoding high-affinity transporters (e.g., *pst* and *phn*) and low-affinity transporters (e.g., *pit*) mediate Pi uptake under P-deficient and P-replete soil conditions, respectively (Hsieh and Wanner, 2010). Genes associated with PSR regulation, including *phoU, phoR, phoP*, and *phoB*, enable microorganisms to maintain a certain P content under P-limited conditions (Devine, 2018; Hsieh and Wanner, 2010; Tee et al., 2021). Several studies have found that different P input levels significantly affect functional genes related to soil P cycling; however, the potential influence of P-fertilizer types remains unclear.

To address this gap, this study focused on a maize cropping system in the black soil region of Northeast China. Based on an 8-year spatially stratified experiment, we investigated the response of soil microorganisms to stratified P application, with the following objectives: 1) to identify the optimal P-fertilizer type suitable for stratified application, 2) to investigate the effects of long-term stratified fertilization on soil microbial diversity and functionality, and 3) to uncover the microbial mechanisms underlying enhanced maize yield. Based on the experimental findings, we further aimed to establish a theoretical framework for optimizing fertilization practices and yield-enhancing strategies in sustainable agricultural systems.

## 2 Materials and methods

### 2.1 Site description

The experiment was carried out at the Experimental Station of Jilin University, Changchun, China (125°14′ E, 43°56′ N) since 2017. The site has a frost-free period of 140 days and an average annual effective accumulated temperature of 3442.3 °C. The soil type is classified as black calcic soil. The initial soil properties (0–20 cm) before the experiment were as follows: pH 5.81; organic matter content 2.99%; total nitrogen (TN) 0.67 g kg^-1^; total phosphorus (TP) 0.35 g kg^-1^; total potassium (TK) 9.75 g kg^-1^; available nitrogen 105.48 mg kg^-1^; available phosphorus (AP) 16.75 mg kg^-1^; available potassium 140.37 mg kg^-1^; bulk density 1.23 g cm^-3^; and soil temperature 14.8 °C.

### 2.2 Experimental design

The maize cultivar ‘Xianyu 335’ was used in the field experiment with a planting density of 75,000 plants per hectare. The planting arrangement followed uniform spacing with 65-cm row spacing. Each experimental plot had an area of 39 m² (3.9 m × 10 m). A balanced compound fertilizer (N-P-K = 15-15-15) was applied as the basal fertilizer (20 cm below the soil surface) at the sowing stage. Three different starter P fertilizers were selected for comparison, which were placed 5 cm laterally and below the seed as a band during sowing (Randall and Hoeft, 1988): ammonium polyphosphate (APP), monoammonium phosphate (MAP), and calcium magnesium phosphate (CMP). Preston et al. (2019) demonstrated that demonstrated that co-application of starter fertilizers with broadcast or deep-banded P fertilizer enhances phosphorus uptake and can increase corn yields, particularly when the starter-P proportion exceeds 20%. Additional urea and potassium sulfate were top-dressed at the jointing stage to achieve a uniform N application rate up to 200 kg N ha^-1^, while the total K application was maintained at 90 kg K_2_O ha^-1^. Accordingly, five P-fertilizer treatments were established for comparison as follows: CK (without P application), FP (75 kg P_2_O_5_ ha^-1^ as basal fertilizer), APP (60 kg P_2_O_5_ ha^-1^ as basal fertilizer + 15 kg P_2_O_5_ ha^-1^ starter P), MAP (60 kg P_2_O_5_ ha^-1^ as basal fertilizer + 15 kg P_2_O_5_ ha^-1^ starter P), and CMP (60 kg P_2_O_5_ ha^-1^ as basal fertilizer + 15 kg P_2_O_5_ ha^-1^ starter P) (**Supplementary Table S1**; **Supplementary Figure S1**). The experimental protocol followed an annual cycle with sowing in May and harvesting in October, during which both soil and maize plant samples were collected for further analysis.

### 2.3 Sample collection

From 2017 to 2024, mature maize plant samples were collected annually, including both aboveground and belowground parts. The yield data were standardized and converted to per-hectare values based on the harvested plot area. All plant samples were oven-dried at 65°C to constant weight for subsequent determination of plant nutritional components. Additionally, the following yield components were measured: ear length, ear diameter, kernel number per ear, ear tip length, and 100-kernel weight. The seed moisture content was determined in triplicate using a PM-8188 moisture meter (PM-8188-A, KETT, Tokyo, Japan). All final yield data were standardized to the 14% seed moisture content.

In October 2024, rhizosphere soil samples (0–20 cm depth) were collected from each experimental plot, with three replicate soil samples obtained per plot to ensure representative sampling. First, three maize plants with intact root systems were randomly selected from each plot. Subsequently, rhizosphere soil was collected by carefully removing the tightly adhering soil from root surfaces using sterile brushes (Riley and Barber, 1969). Each soil sample was passed through a 2-mm sieve to remove large root fragments, followed by division into two aliquots: one aliquot was preserved for microbial sequencing analysis and the other was used for physicochemical characterization.

### 2.4 DNA extraction and sequencing

The total DNA was extracted from each homogenized soil sample E.Z.N.A™ Mag-Bind Soil DNA Kit (Omega, M5635-02, USA). The V3-V4 region of bacterial 16S rRNA gene was amplified based on the primers 338F/806R (Caporaso et al., 2012), ITS primers ITS1 and ITS2 were used for fungal DNA amplification (Mueller et al., 2014). Samples were delivered to Sangon BioTech (Shanghai, China) for library construction using universal Illumina adaptor and index. Sequencing was performed using the Illumina MiSeq system (Illumina MiSeq, USA), according to the standard protocols. Then, the raw sequences were quality filtered, merged, and analyzed by standard methods. Finally, operational taxonomic units (OTUs) were clustered at 97% similarity cutoff using Usearch software (version 11.0.667). Bacterial and fungal OTU representative sequences were classified taxonomically by blasting against the RDP Database and UNITE fungal ITS Database, respectively.

### 2.5 Statistical analysis

Statistical analysis was performed using IBM SPSS Statistics 27.0.1 (IBM Corp., Armonk, NY, USA). One-way analysis of variance was conducted to assess the effects of different P fertilizers on soil physicochemical properties, plant nutrient contents, and crop yield parameters. Treatment means were compared using Fisher’s least-significant difference *post-hoc* test at a significance level of α = 0.05. Several agronomic indices were calculated using standard formulas.

PUE, based on the P-fertilizer application rate and aboveground P uptake, was calculated as follows (Margenot et al., 2025) (Equation 1):


(1) $$PUE\;(\%)=\frac{P\;uptake,\;fertilized-P\;uptake,\;control\;}{P\;applied}\times 100$$

where aboveground P uptake refers to the total P absorption in plant straw and grains.

The partial factor productivity of phosphorus (PFP, kg kg^-1^) index serves as a crucial indicator for evaluating phosphorus fertilizer use efficiency (Yang et al., 2022), reflecting the crop yield obtained per unit of P-fertilizer input, which was calculated with the following formula (Equation 2):


(2) $$PFP\;=\frac{Y\;fertilized}{P\;applied}$$

Agronomic efficiency of phosphorus (AEP) quantifies the incremental crop yield per unit of P fertilizer applied, reflecting the actual contribution of fertilization to yield enhancement (Hartina et al., 2025), which was calculated with the following formula (Equation 3):


(3) $$AEP\;(\%)=\frac{Y\;fertilized-Y\;control}{P\;applied}\times 100$$

Finally, the P surplus (kg ha^-1^) was calculated according to Equation 4 below (Guan et al., 2022), which refers to the portion of total P input exceeding the total output in an agricultural system, indicating either soil P accumulation or potential environmental risks. As a key indicator for evaluating the sustainability of phosphorus management, P surplus is widely used to assess the P-cycling status in agroecosystems.


(4) P surplus=P applied−P uptake

Analyses of microbial α- and β-diversity were conducted using RStudio. After performing significance analysis on the 16S sequencing data, the *P*-values were corrected for multiple hypothesis testing using the FDR (False Discovery Rate) method. Principal coordinate analysis (PCoA) was employed to visually depict the compositional differences of rhizosphere bacterial and fungal communities across different P fertilizer treatments. Permutational multivariate analysis of variance (PERMANOVA) was further used to test for significant differences in community composition among treatments. To visualize associations between P fertilizer application and key rhizosphere microbial taxa, as well as soil functional genes, GraphPad Prism 8 (GraphPad Software, San Diego, CA, USA) was utilized. STAMP (Statistical Analysis of Metagenomic Profiles) was applied to identify significantly differentially abundant species under varying water transport conditions and characterize their preferential environmental correlations.

Functional profiling was performed using default parameters in FUNGuild (Fungal Functional Guild), PICRUSt2, and the KEGG database. Genes associated with soil P-cycling were defined as soil functional genes following previously reported methods (Dai et al., 2020; Hsieh and Wanner, 2010; Liu et al., 2023b; Mahmood et al., 2020; McGrath et al., 2013). For metagenomic data processing, additional methodological details are provided in Appendix S2.

Finally, structural equation modeling (SEM) was used to quantify both direct and indirect effects of soil microbial communities, soil physicochemical properties, functional gene abundances, phosphorus fertilizer uptake/utilization efficiency, and stratified P fertilization on the sustainability of maize yield.

## 3 Results

### 3.1 Soil physical properties and plant nutrient content

APP resulted in the highest values of soil bulk density, water content, and electrical conductivity, as well as the greatest solid-to-liquid ratio among the five experimental groups. CMP exhibited the highest soil pH value among all treatments; APP and MAP treatments resulted in relatively lower soil pH values, rendering the soils acidic (**Table 1**). CMP demonstrated the highest air ratio (30.25%) among the four fertilization treatments, indicating improved soil aeration and higher porosity, which are conducive to plant root growth and soil microbial activity. CMP also resulted in the lowest bulk density value among the various fertilization conditions, demonstrating a significant improvement in soil aeration and water infiltration capacity compared to those of APP.

**Table 1:** Effects of different P fertilizers on soil structure and physical properties. Different lowercase letters in each rows indicate significant differences (P < 0.05) among different P fertilizer treatments, the same below.

Items	CK	FP	APP	MAP	CMP
Soil Bulk Density (g cm^-3^)	1.44 ± 0.1**b**	1.46 ± 0.11**ab**	1.58 ± 0.11**a**	1.52 ± 0.12**ab**	1.45 ± 0.14**b**
Soil Moisture (%)	13.62 ± 0.91**c**	14.7 ± 0.71**ab**	15.21 ± 1.31**a**	14.52 ± 0.79**abc**	13.96 ± 0.96**bc**
Soil porosity (%)	50.61 ± 3.76	50.36 ± 4.05	46.86 ± 5.36	47.9 ± 4.62	50.5 ± 4.52
True Density (g cm^-3^)	2.52 ± 0.06	2.52 ± 0.02	2.53 ± 0.10	2.5 ± 0.03	2.51 ± 0.02
pH	5.93 ± 0.10**ab**	6.13 ± 0.16**a**	5.48 ± 0.46**c**	5.66 ± 0.37**bc**	6.14 ± 0.12**a**
Electrical Conductivity (μS cm^-1^)	53.83 ± 8.38**b**	69.17 ± 7.25**b**	119.17 ± 47.55**a**	76.33 ± 53.63**ab**	90.33 ± 14.84**ab**
Air Ratio (%)	30.99 ± 6.03**a**	28.9 ± 4.97**ab**	22.83 ± 5.62**b**	25.84 ± 5.79**ab**	30.25 ± 7.23**a**
Solid Ratio (%)	49.39 ± 3.76	49.64 ± 4.05	53.14 ± 5.36	52.1 ± 4.62	49.5 ± 4.52
Liquid Ratio (%)	19.62 ± 2.5**c**	21.46 ± 1.29**bc**	24.02 ± 1.91**a**	22.06 ± 1.61**ab**	20.25 ± 2.95**bc**
Void Ratio (%)	15.78 ± 1.21	17.24 ± 0.97	17.96 ± 1.84	16.99 ± 1.08	16.24 ± 1.3
Water-Solid-Ratio (%)	0.4 ± 0.03**b**	0.43 ± 0.03**ab**	0.46 ± 0.07**a**	0.43 ± 0.03**ab**	0.41 ± 0.04**b**
Saturation Percentage (%)	39.22 ± 7.27**c**	43 ± 5.76**bc**	51.89 ± 7.35**a**	46.64 ± 7.17**ab**	40.77 ± 8.82**bc**

**Figure 1:**
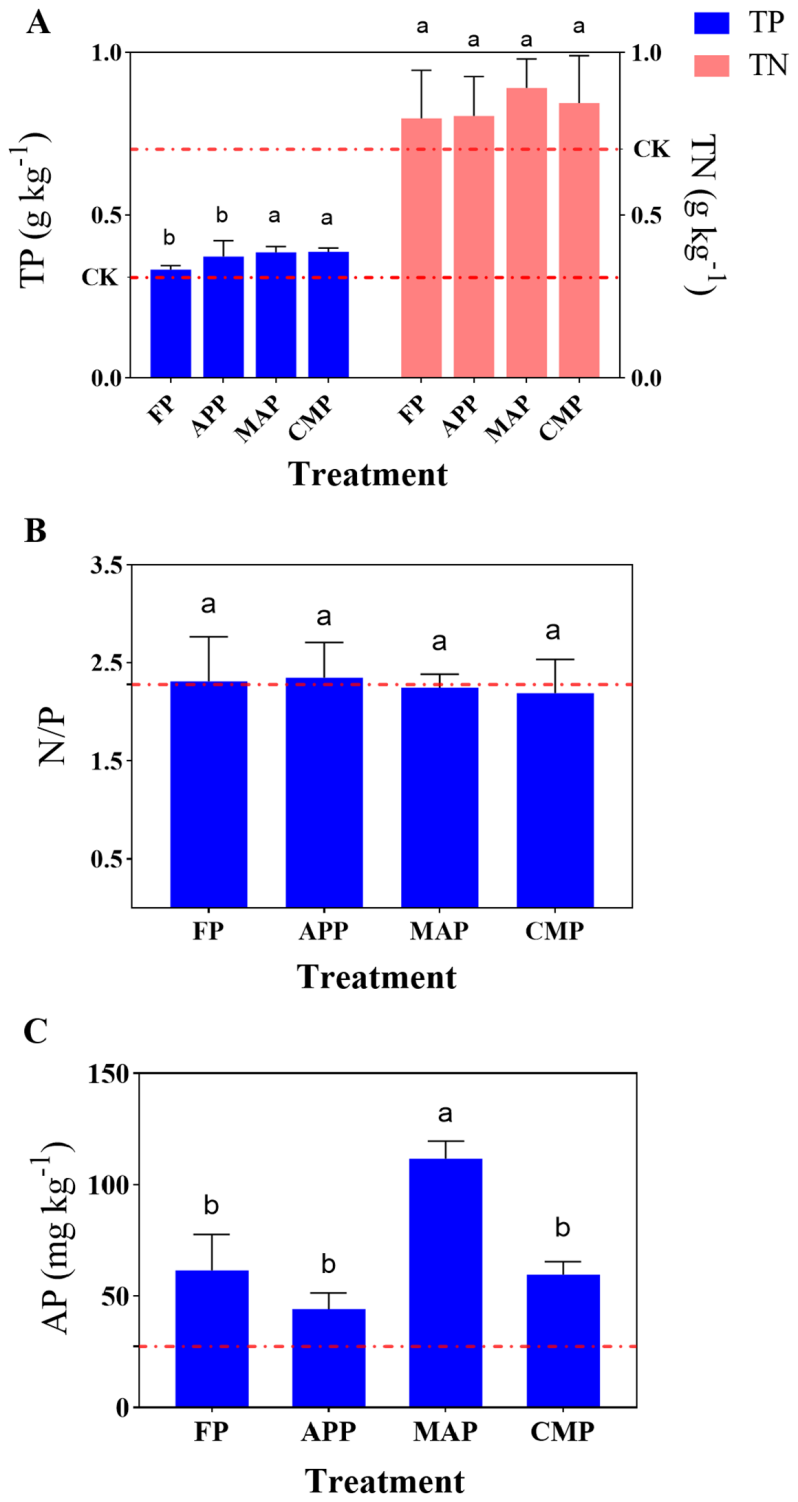
Effects of different P fertilizers on TN & TP **(A)**, N/P ratio **(B)**, and AP **(C)** content in soil. Error bars represent SD values of the means (n=3). Different lowercase letters indicate significant differences (*P* < 0.05) among different P fertilizer treatments; the control treatment was marked with a red dashed line, the same below.

All P-fertilizer treatments except FP showed a significantly higher total P (TP) content compared to that of the CK group. Specifically, APP, MAP, and CMP increased the TP content by 3.60%, 18.62%, and 15.62%, respectively, relative to that of FP, while FP resulted in a 7.42% increase in TP over that of the CK treatment. Notably, MAP and CMP demonstrated the highest TP values among all treatments. The total N (TN) content under the MAP treatment was 11.53% higher than that under FP, indicating that MAP exhibited the most pronounced effect on enhancing the soil N and P contents. Although MAP showed the highest TN value among all treatments, the difference was not statistically significant (**Figure 1A**). APP had the highest stoichiometric N/P ratio of 2.35 among all treatments, although no significant differences were observed across the five treatment groups (**Figure 1B**). MAP demonstrated the most significant enhancement in soil AP content, showing an 81.46% increase (*P* < 0.0001) compared to that of the FP group. In contrast, both the APP and CMP applications resulted in decreased AP levels compared to that of the FP condition (**Figure 1C**). These results collectively indicate that MAP outperformed the other three fertilizer types in improving soil nutrient content, particularly with respect to the AP concentration.

**Figure 2:**
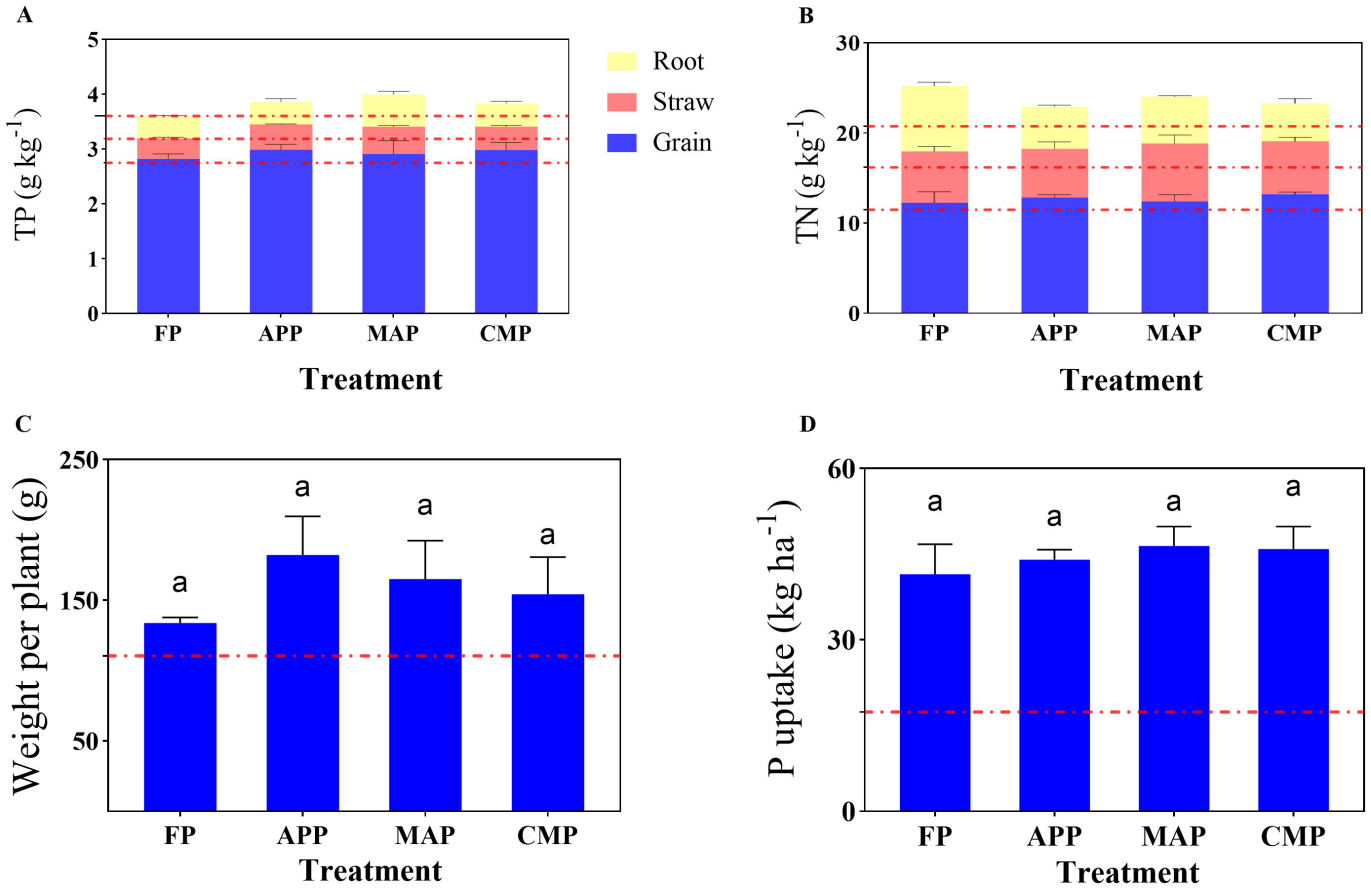
Effects of different P fertilizers on TP **(A)** and TN **(B)** contents in various maize plant parts, as well as shoot dry weight per plant **(C)** and P uptake per plant **(D)**.

The TP content in maize straw under the MAP treatment differed significantly from that of the FP, and CMP groups, with increases of 33.78% (*P <* 0.0001), and 16.47% (*P =* 0.0054), respectively. Additionally, the maize straw of the APP and CMP groups exhibited 24.32% and 14.86% higher TP levels than that of the FP group. The TP content in maize grains followed the order APP > CMP > MAP > FP. In the root systems, compared to that of FP, MAP increased the TP content by 47.5% (*P =* 0.0002), while CMP showed a 6.25% reduction. Notably, FP exhibited a 2.44% decrease in TP content relative to CK (**Figure 2A**). The TN content in maize straw showed a 12.61% increase under the MAP treatment compared to FP, while CMP demonstrated a 2.80% elevation relative to that of the FP treatment. In the root systems, FP exhibited a 55.58% higher TN content than that of the APP treatment (*P* < 0.0001) (**Figure 2B**). Comparative analysis of nutrient accumulation across different plant parts revealed a consistent pattern for both TN and TP: grains > straw > roots.

At maturity, although the APP treatment resulted in a 36.07% increase in dry weight compared to that under FP, the difference was not statistically significant. Similarly, MAP increased the dry weight by 23.23% relative to that of the FP condition, but neither difference reached statistical significance (**Figure 2C**). All four P-fertilizer treatments resulted in significantly higher total P uptake (calculated as the sum of P uptake in the grains and straw) compared to that of the CK control group. Specifically, APP, MAP, and CMP showed increases of 6.08%, 11.91%, and 10.54% in total P uptake, respectively, compared to that of the FP treatment (**Figure 2D**).

### 3.2 Maize yield and P-efficiency parameters

**Figure 3:**
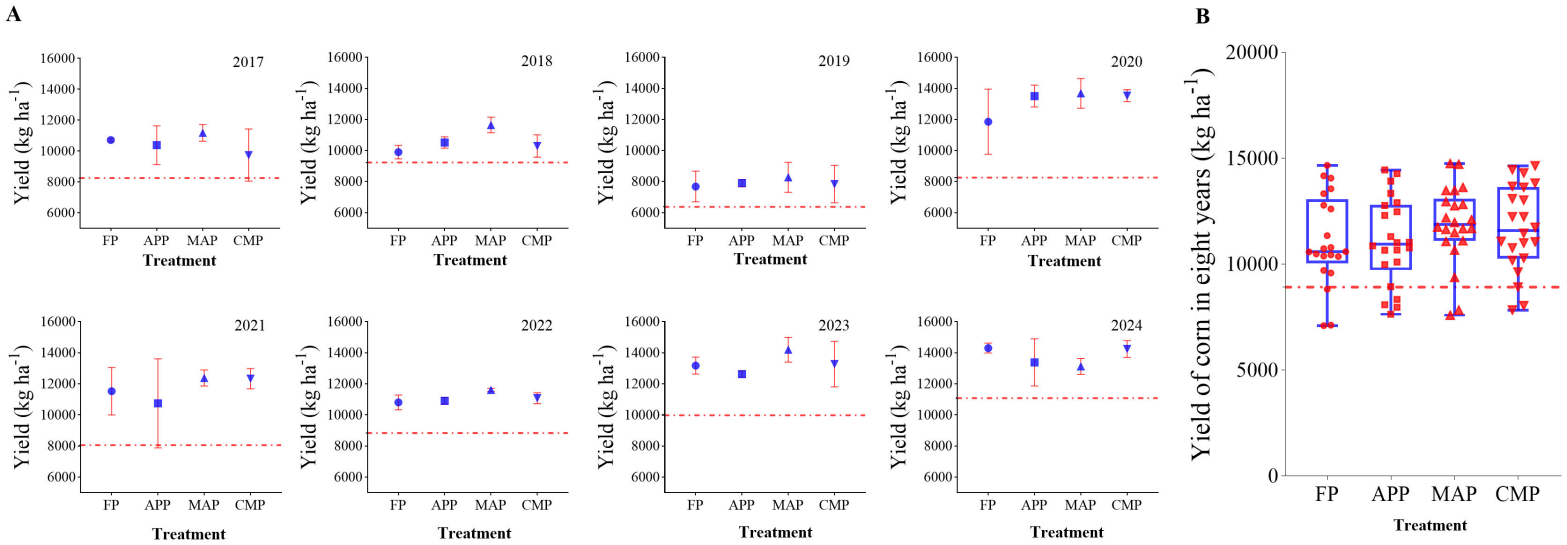
Yield differences among treatments during 2017-2024 **(A)** and average yield of all treatments **(B)**.

Integration of the 8-year yield data showed substantial interannual variability in maize production. The yield decline in 2019 was primarily attributed to typhoon-induced lodging. Due to the potential influence of the 2019 typhoon on yield measurements—which may have introduced anomalies—the comprehensive yield variability after excluding 2019 (presented in **Supplementary Figure S2**). Both the original and adjusted datasets exhibit identical trends. Annual yields clustered within the range of 8,000–12,000 kg ha^-1^ during 2017 and 2018, but reached higher values of 10,000–14,000 kg/ha in 2023 and 2024. Collectively, the dataset demonstrates a general upward yield trend across the observation period (**Figure 3A**). The mean annual yields across treatments ranked in descending order as follows: MAP (11,859 kg ha^-1^), CMP (11,691 kg ha^-1^), FP (11,110 kg ha^-1^), APP (11,086 kg ha^-1^), and CK (8,924 kg ha^-1^). All four P-fertilized treatments showed significantly higher yields than the non-fertilized treatment (CK) (*P* < 0.01). Specifically, MAP and CMP exhibited 6.74% and 5.23% increases relative to FP, respectively, although these differences were not statistically significant (**Figure 3B**).

The starter fertilizers demonstrated superior P-efficiency indices compared to FP. Specifically, MAP exhibited the highest PFP and AEP, showing 4.07% and 16% increases over those of FP, respectively. Furthermore, long-term application of starter fertilizers (APP, MAP, CMP) resulted in a lower soil P surplus than that of the FP treatment, with reductions of 7.52%, 14.74%, and 13.04%, respectively. Moreover, the PUE increased by 19.43%, 38.06%, and 33.68% in the APP, MAP, and CMP treatments compared to that of the FP group (**Table 2**). These results demonstrate that under equivalent P application rates, the addition of starter fertilizers enhances PUE and reduces the soil P surplus.

**Table 2:** Response of P-nutrient efficiency indicators to P fertilizer types and application methods.

Items	FP	APP	MAP	CMP
PUE (%)	17.34 ± 7.00	20.71 ± 2.38	23.94 ± 4.50	23.18 ± 5.31
PFP (kg kg^-1^)	178.41 ± 18.11	168.36 ± 2.00	185.68 ± 9.82	181.98 ± 16.29
AEP(%)	45.38 ± 18.11	35.32 ± 2.00	52.64 ± 9.82	48.94 ± 16.29
P Surplus (kg ha^-1^)	33.52 ± 5.25	31.00 ± 1.78	28.58 ± 3.38	29.15 ± 3.98

### 3.3 Microbial community structure and composition

**Figure 4:**
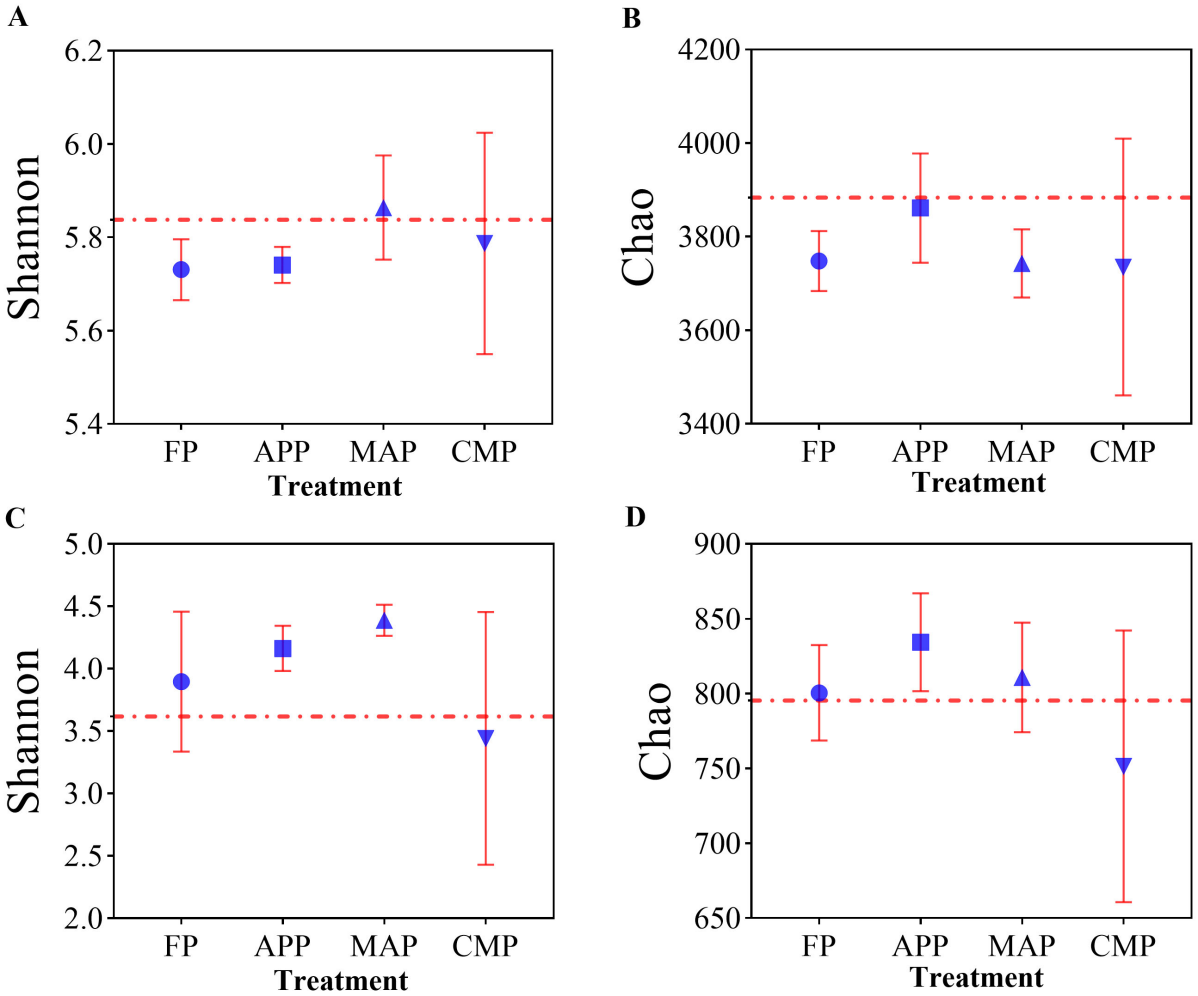
Effects of different P fertilizers on the diversity of soil bacterial **(A, B)** and fungal **(C, D)** communities.

Alpha-diversity analysis revealed significant differences among P-fertilization methods and types. In the bacterial communities of the phosphorus-amended treatments, the Shannon index of the MAP treatment was comparable to that of the CK control, whereas the FP treatment exhibited the lowest value. Specifically, the Shannon index of MAP was 2.27% higher than that of FP and 2.09% higher than APP. The CMP treatment showed a 1.05% increase over FP (**Figure 4A**). For the Chao index, all phosphorus-amended treatments were lower than the non-phosphorus control (CK). Among these, APP had the highest Chao index, exceeding MAP by 3.17% and FP by 3.02% (**Figure 4B**). In the fungal communities, MAP displayed the highest Shannon index, which was 27.62% greater than that of CMP and 12.56% higher than FP (**Figure 4C**). The Chao index of APP was 11.03% higher than CMP, while MAP exceeded CMP by 7.90% (**Figure 4D**).

**Figure 5:**
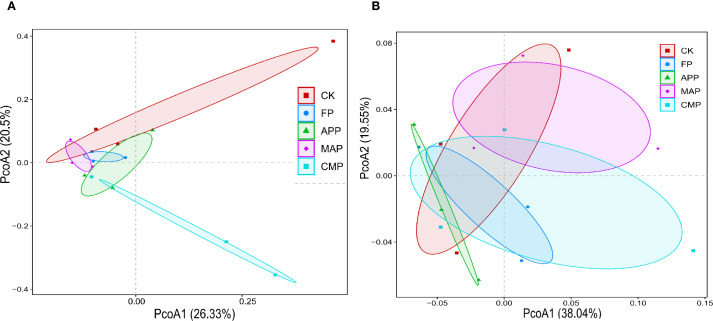
Effects of different treatments on the composition of soil fungal **(A)** and bacterial **(B)** communities.

Beta-diversity of soil fungal and bacterial communities was assessed by calculating Bray–Curtis dissimilarity matrices and the results were visualized using principal coordinate analysis (PCoA), with the first two axes (PCoA1 and PCoA2) explaining 26.33% and 20.5% (fungal communities, **Figure 5A**) or 38.04% and 19.55% (bacterial communities, **Figure 5B**) of the total variance, respectively. Fungal communities formed discrete clusters, reflecting their distinct compositional profiles, whereas bacterial communities exhibited pronounced clustering patterns, indicating partial community restructuring. PERMANOVA further revealed significant differences in β-diversity among treatments for fungal communities (*F* = 1.56, *P* = 0.017), with relatively independent distribution patterns across fertilization regimes. In contrast, bacterial communities showed no significant β-diversity variation among treatments (*F* = 0.84, *P* = 0.677).

**Figure 6:**
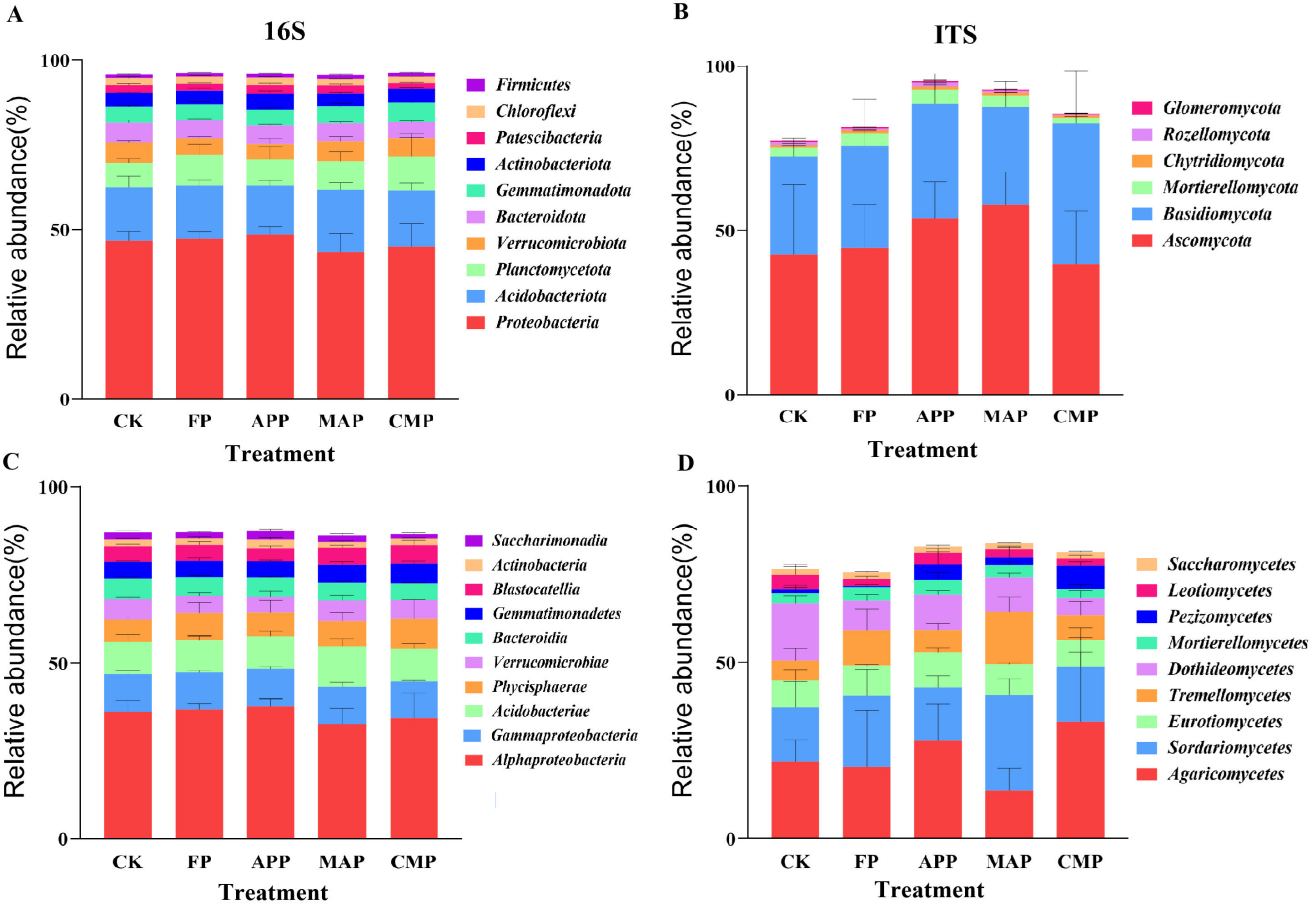
Relative abundance of dominant species at the phylum **(A, B)** and class **(C, D)** taxonomic level.

Excluding unclassified phyla, the top 10 most abundant bacterial and fungal phyla across all soil samples were selected to investigate the impacts of different P application regimes on soil microbiological communities. In the FP, APP, MAP, and CMP treatments, the phyla Ascomycota, Basidiomycota, and Mortierellomycota accounted for 79.52%, 92.66%, 90.98%, and 84.27% of the total relative abundance, respectively. Both APP and MAP significantly enhanced the relative abundance of Ascomycota by 20.34% and 29.52%, respectively, compared to that detected under the FP treatment. Concurrently, APP and CMP also increased the relative abundance of Basidiomycota by 11.95% and 37.54%, respectively. The dominant bacterial phyla across all soil samples were Proteobacteria, Acidobacteriota, and Planctomycetota, collectively accounting for 71.85%, 70.67%, 70.06%, and 71.43% of the total relative abundance in the FP, APP, MAP, and CMP treatments, respectively. Notably, MAP significantly increased the relative abundance of Acidobacteriota by 16.82% compared to that under FP (*P* < 0.05), while CMP showed a 5.72% increase in Planctomycetota abundance relative to that of the FP treatment (**Figure 6A**).

The dominant fungal classes across all soil samples were consistently Agaricomycetes, Sordariomycetes, and Eurotiomycetes, collectively representing 49.00%, 52.78%, 49.50%, and 56.20% of the total relative abundance in the FP, APP, MAP, and CMP treatments, respectively. Notably, compared to FP, MAP induced the greatest increases in relative abundance for both Sordariomycetes (34.53%) and Tremellomycetes (48.16%) (**Figure 6D**). Sordariomycetes, a phylogenetically significant fungal class within Ascomycota, plays dual ecological roles as key decomposers and potential pathogens. In the soil bacterial communities, Acidobacteriae exhibited a 23.13% increase, whereas Alphaproteobacteria showed an 11.22% reduction under the MAP treatment compared to FP. Integrated analysis of the relative abundance stacked plots revealed that different P-fertilization treatments exerted only a minimal influence on the composition of dominant microbial taxa at both the phylum and class levels. However, significant variations were observed in the relative abundance proportions of these taxa. Notably, fungal communities demonstrated greater sensitivity to P fertilizers and application methods than bacterial communities.

**Figure 7:**
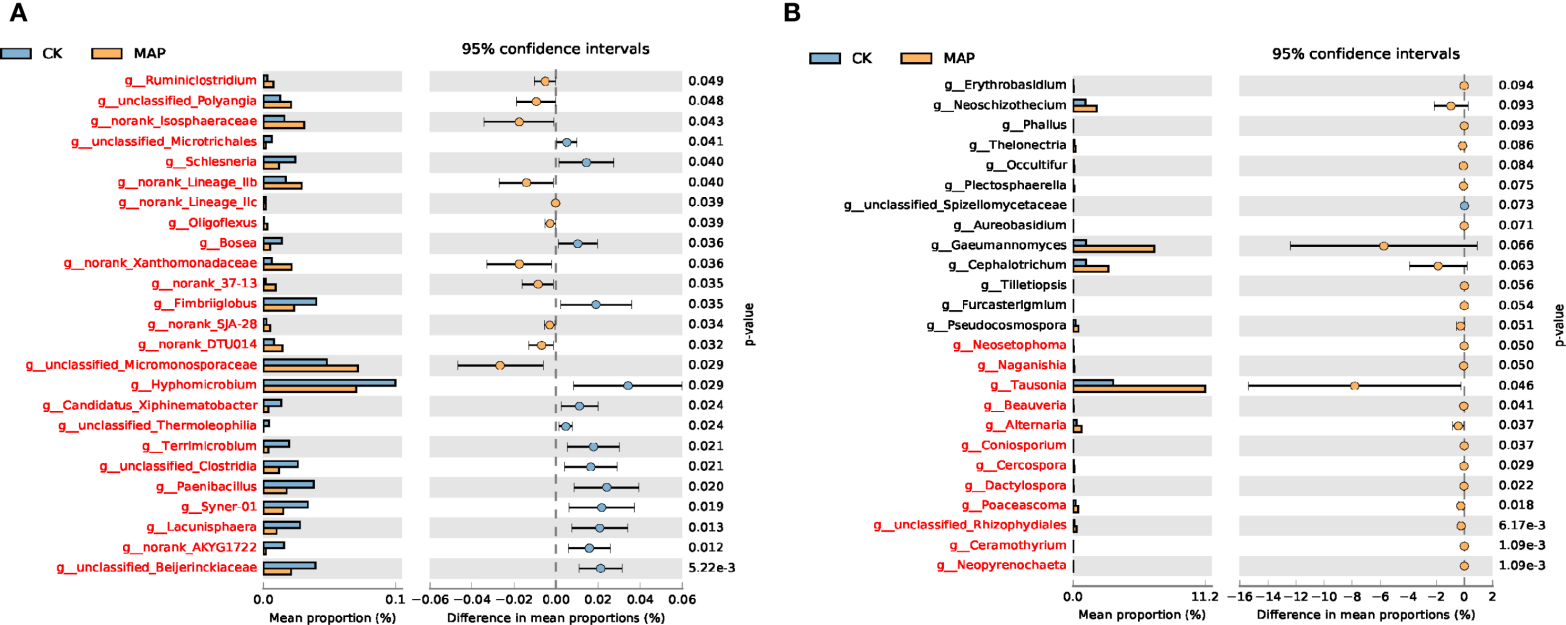
Comparative analysis of dominant genera-level taxa in bacterial **(A)** and fungal **(B)** communities based on relative abundance.

To investigate the interactions between P fertilizers and soil microbial communities, based on our observations of the relatively greater efficacy of the MAP treatment in enhancing maize yield, we conducted STAMP analysis to compare genus-level relative abundance differences between the MAP and CK treatments. Statistically significant differential taxa were identified through this analysis, along with their environmental preference trends. At the genus level, bacterial communities exhibited significant differences across all detected taxa between MAP and CK treatments (**Figure 7A**), whereas fungal communities showed limited differences in abundance between these two treatments. This demonstrates that MAP fertilization exerts stronger selective pressure on the bacterial community structure than on fungal assemblages. Notably, the fungal genus *Tausonia* exhibited significantly higher relative abundance in MAP soil compared to CK soil (*P* = 0.046) (**Figure 7B**).

**Figure 8:**
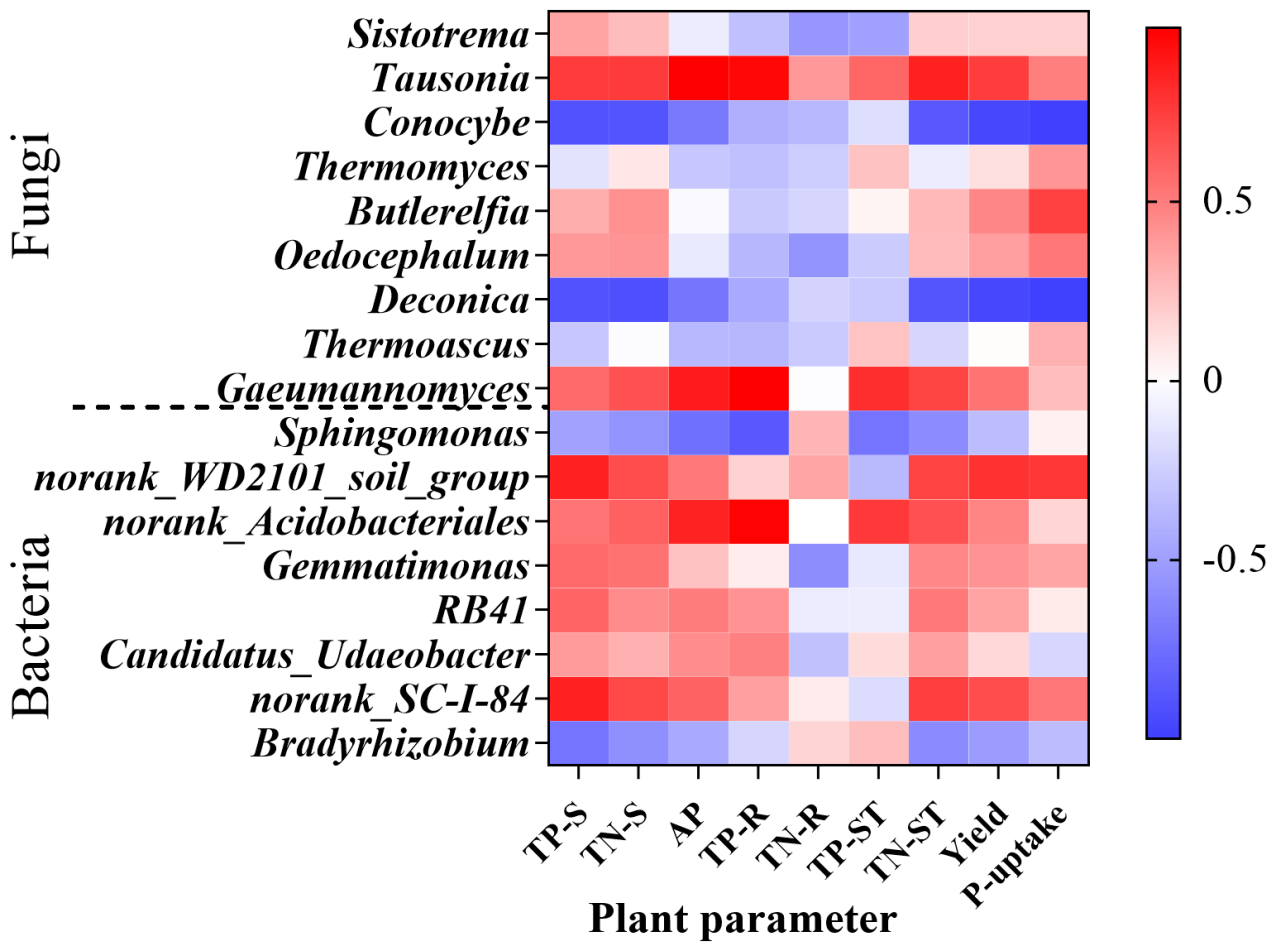
Heatmap of genus-level soil microbial correlations with edaphic and plant parameters. AP, soil Olsen-P content; TP-S, the total phosphorus of soil; TN-S, the total nitrogen of soil; TP-R, the total phosphorus of root; TN-R, the total nitrogen of root; TP-ST, the total phosphorus of straw; TN-ST, the total nitrogen of straw. The same below.

Correlation analysis between dominant soil microbial genera (bacteria and fungi) and soil/plant nutrient parameters revealed key functional relationships with crop yield. *Tausonia* exhibited significant positive correlations with AP (*P* = 0.003) and TP-R (*P* = 0.012); the fungal genus *Deconica* showed significant negative correlations with TN-S (*P* = 0.043), maize yield (*P* = 0.015), and P-uptake (*P* < 0.0001); the fungal genus *Gaeumannomyces* demonstrated a significant positive correlation with TP-R (*P* = 0.002); and the bacterial lineage *norank_Acidobacteriales* exhibited a significant positive correlation with TP-R (*P* = 0.006) (**Figure 8**)).

### 3.4 Soil microbial functional prediction and P-cycling functional genes

To identify distinct functional groups within rhizosphere soil fungal communities and link their relative abundances to different P-application methods and types, we classified fungal operational taxonomic units into specific trophic groups, which were then further subdivided into defined ecological guilds. Using the FUNGuild framework, soil fungal communities were primarily categorized into three major trophic modes: saprotrophy, symbiotrophy, and pathotrophy. Based on these trophic classifications, fungi were additionally assigned to multiple ecological guilds (e.g., animal pathogens, endophytes, mycorrhizal fungi).

**Figure 9:**
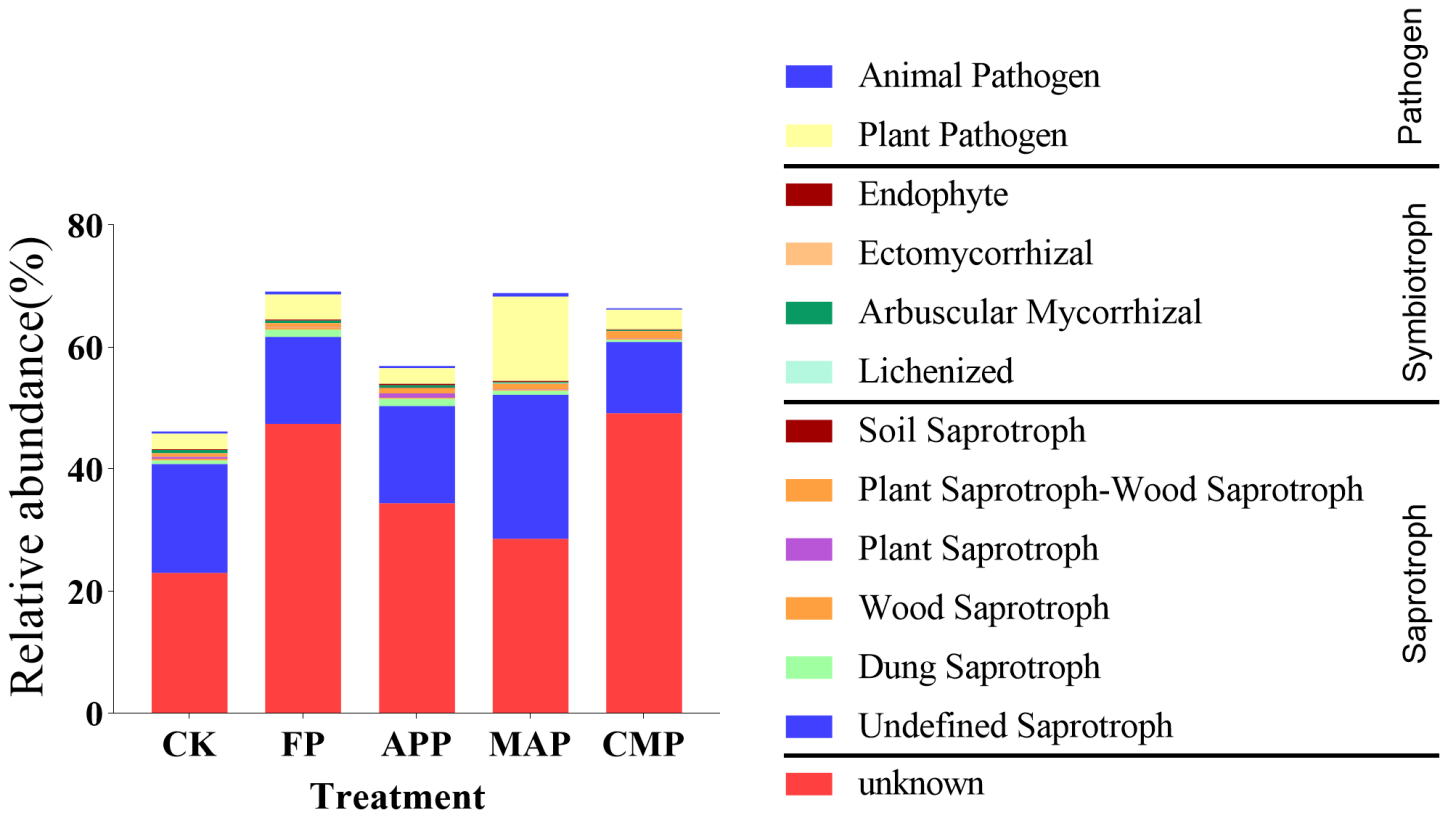
Functional classification of fungal communities under different P fertilization treatments.

Overall, the groups “Undefined Saprotrophs” and “Plant Pathogens” exhibited high relative abundances across all treatments. Among these, the MAP treatment displayed the highest values: Undefined Saprotrophs showed increases of 32.32% and 65.01% relative to the abundance of this group in the CK and FP conditions, respectively. In contrast, Plant Pathogens displayed a marked increase, with relative abundances rising by 440.78% and 432.43% relative to those under CK (*P* = 0.0406) and APP (*P* = 0.0412), respectively (**Figure 9**). These findings suggest that MAP strengthens the functional role of saprotrophic fungi within the fungal community, exhibiting superior efficiency in organic matter decomposition and nutrient cycling compared to other P fertilizers. However, this treatment also elevated the relative abundance of plant pathogens, posing a potential risk of plant disease.

**Figure 10:**
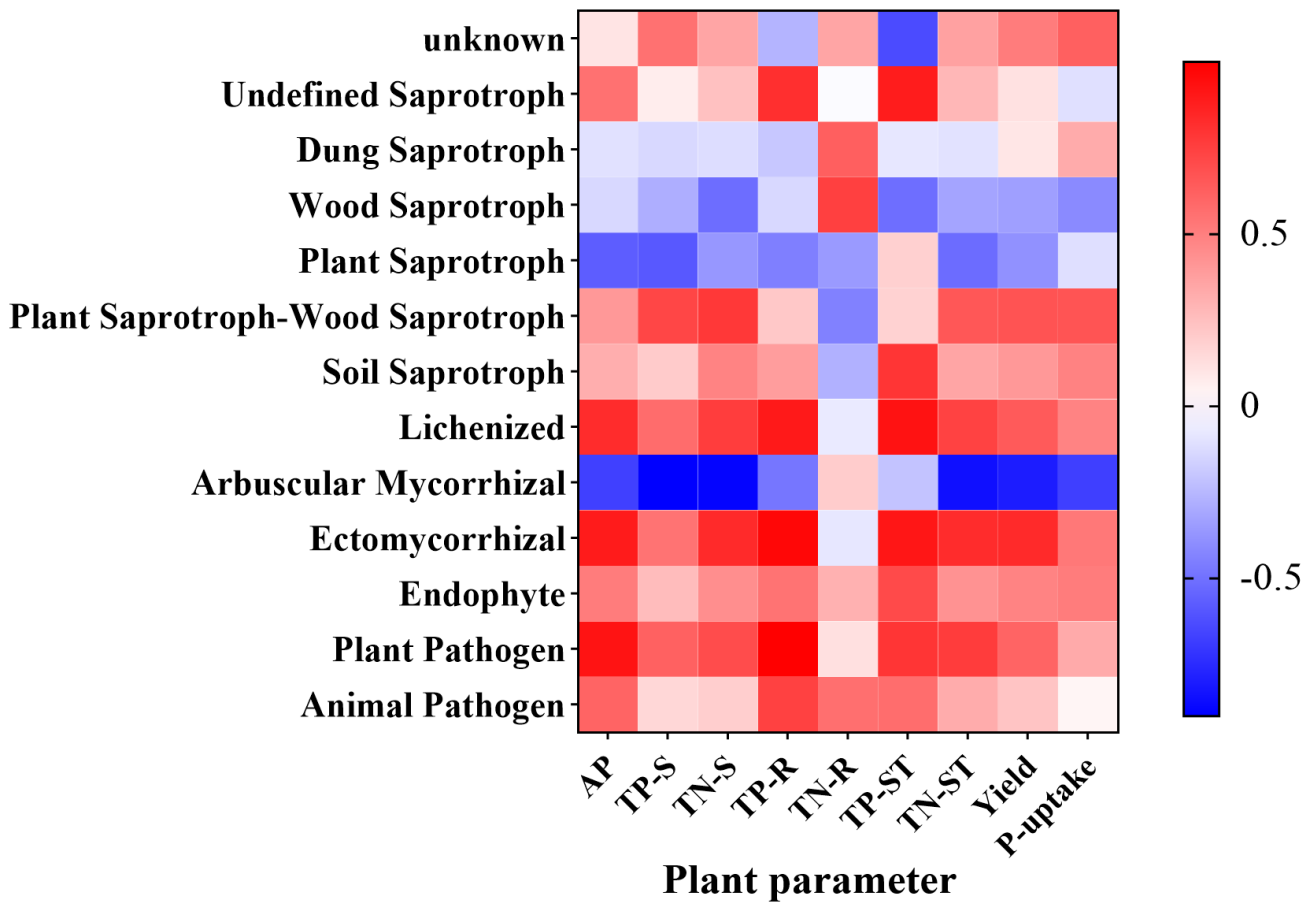
Heatmap illustrating the correlations between soil fungal functions and various indicators.

The abundance of Undefined Saprotrophs showed a positive correlation with TP-ST (*P* = 0.045). The abundance of the Lichenized group exhibited positive correlations with TP-R (*P* = 0.037) and TP-ST (*P* = 0.024). Arbuscular Mycorrhizal fungi were negatively correlated with TN-S (*P* = 0.044) and TP-S (*P* = 0.037). Ectomycorrhizal fungi demonstrated a positive correlation with TP-R (*P* = 0.032), while Plant Pathogens were positively associated with AP (*P* = 0.026) and TP-R (*P* < 0.0001) (**Figure 10**).

**Figure 11:**
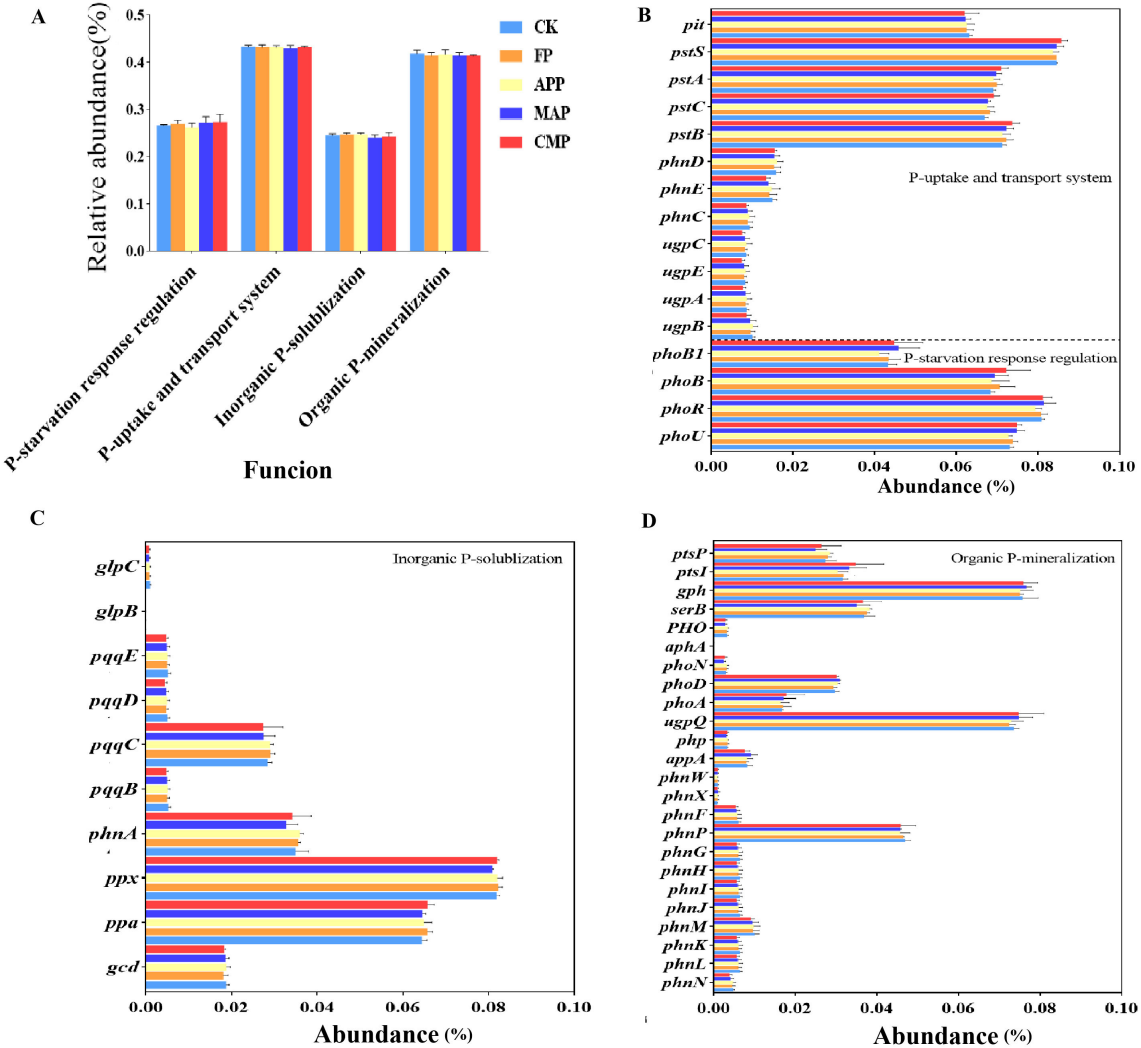
Relative abundance of representative P-cycling related genes in bacterial communities under different P fertilizer treatments.

Based on previous studies, a total of 50 soil P cycling-related genes and their corresponding Kyoto Encyclopedia of Genes and Genomes (KEGG) Orthology (KO) annotations were selected (Dai et al., 2019; Hsieh and Wanner, 2010; Liu et al., 2023b; Mahmood et al., 2020; McGrath et al., 2013). Following the functional aggregation of P cycling-related genes, our predictive data analysis revealed that long-term spatially stratified P inputs may exert a negligible impact on the total relative abundance of genes involved in the four functional categories. However, genes associated with the P-uptake and transport system consistently represented the highest proportion among all functional categories (**Figure 11A**). To further explore the differential abundances of P-cycling genes among different P-fertilizer treatments, our visualization analysis revealed that *pstS, phoR, ppx, gph*, and *ugpQ* displayed the highest relative abundances in soil samples. Relative to that of the CK treatment, it is plausible that MAP enhanced the relative abundance of *phoB1*—a gene involved in PSR regulation—by 6.98%, while reducing the abundance of *phnA*—which functions in Pi solubilization—by 5.71%. Compared to FP, MAP further resulted in a 1.35% increase in the relative abundance of *phoU*, a key constituent of the PSR regulatory system. MAP application may enhance the relative abundance of genes encoding enzymes involved in Po mineralization, including *gph*, *appA*, and *phoD*, with their relative abundances increasing by 2.67%, 10.98%, and 6.90%, respectively, compared to those under the FP condition (**Figures 11B–D**). The gene *ugpQ*, which also encodes enzymes involved in Po mineralization, displayed a 2.70% reduction in relative abundance under the FP treatment relative to CK. Compared to that in the FP treatment, our predictive data analysis suggests that MAP and CMP could elevate the relative abundance of *ptsI* by 3.12% and 9.38%, respectively (**Figure 11D**). Concerning genes associated with P uptake and transport, CMP may decrease the relative abundance of the low-affinity phosphate (Pi) transporter gene (*pit*) by 0.32% compared to that found in the FP group (**Figure 11B**).

**Figure 12:**
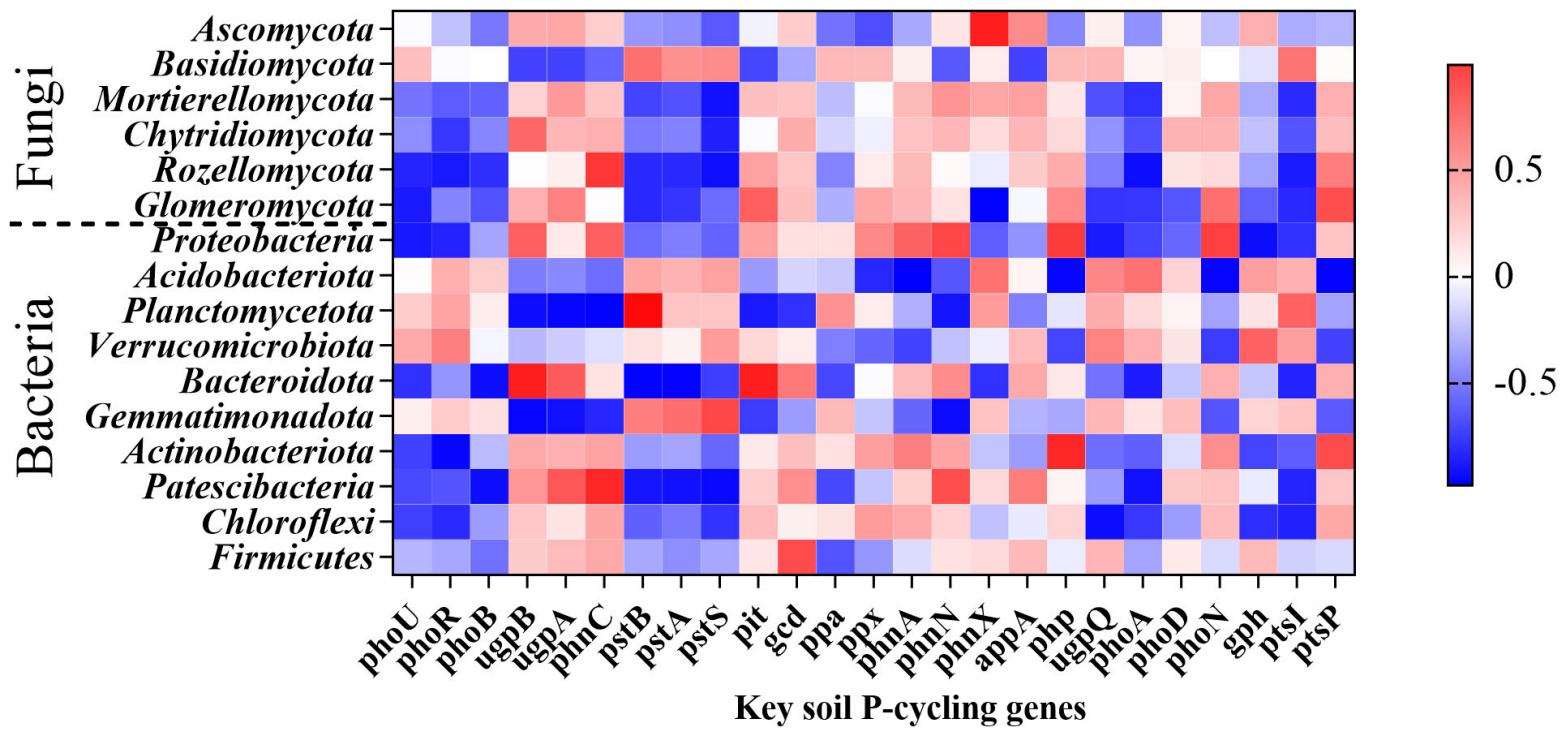
The relationship between major rhizosphere soil microbial taxa (bacteria, fungi) and the relative abundance of key soil functional genes (P-cycling genes).

At the phylum level of fungi, Mortierellomycota showed a significant negative correlation with *pstS* (*P* = 0.029), while Rozellomycota exhibited significant negative correlations with *phoR* (*P* = 0.046)*, pstS* (*P* = 0.025), and *phoA* (*P* = 0.022). Glomeromycota demonstrated significant negative correlations with *phoU* (*P* = 0.049) and *phnX* (*P* = 0.022) but had significant positive correlations with *pit* (*P* = 0.025) and *phnN* (*P* = 0.012). At the bacterial phylum level, Proteobacteria displayed significant positive correlations with *phnA* (*P* = 0.002)*, php* (*P* = 0.036)*, phoN* (*P* = 0.001), and *ptsP* (*P* = 0.001) but exhibited significant negative correlations with *phoU* (*P* = 0.044), *ugpQ* (*P* = 0.048), and *gph* (*P* = 0.023). Acidobacteria showed significant negative correlations with *phnA* (*P* = 0.003)*, php* (*P* = 0.009)*, phoN* (*P* = 0.007), and *ptsP* (*P* = 0.006), whereas there was a significant positive correlation with *gph* (*P* = 0.033). Planctomycetota exhibited significant negative correlations with *ugpB* (*P* = 0.021)*, ugpA* (*P* = 0.009)*, phnC* (*P* = 0.004)*, pit* (*P* = 0.045), and *phnN* (*P* = 0.034) but significant positive correlations with *phoA* (*P* = 0.042)*, pstA* (*P* = 0.001), *pstB* (*P* = 0.005) and *phoB* (*P* = 0.003). Bacteroidota demonstrated significant positive correlations with *ugpB* (*P* = 0.017)*, ugpA* (*P* = 0.011)*, phnC* (*P* = 0.013)*, pit* (*P* = 0.018), and *phnN* (*P* = 0.016) but significant negative correlations with *pstA* (*P* = 0.007)*, pstB* (*P* = 0.005), and *phoB* (*P* = 0.02). Gemmatimonadota exhibited significant negative correlations with *ugpA* (*P* = 0.027)*, ugpB* (*P* = 0.012), and *phnN* (*P* = 0.018), while showing significant positive correlations with *pstB* (*P* = 0.029)*, phoA* (*P* = 0.026), and *ptsI* (*P* = 0.009). Actinobacteriota displayed a significant negative correlation with *phoR* (*P* = 0.01). Patescibacteria showed significant negative correlations with *phoB* (*P* = 0.023)*, pstB* (*P* = 0.036)*, pstA* (*P* = 0.028)*, pstS* (*P* = 0.014), and *phoA* (*P* = 0.028). Firmicutes was significantly positively correlated with *gcd* (*P* = 0.038) (**Figure 12**).

### 3.5 Effects of P fertilization on soil properties, plant growth, rhizosphere microbial community structure and function, and maize yield

**Figure 13:**
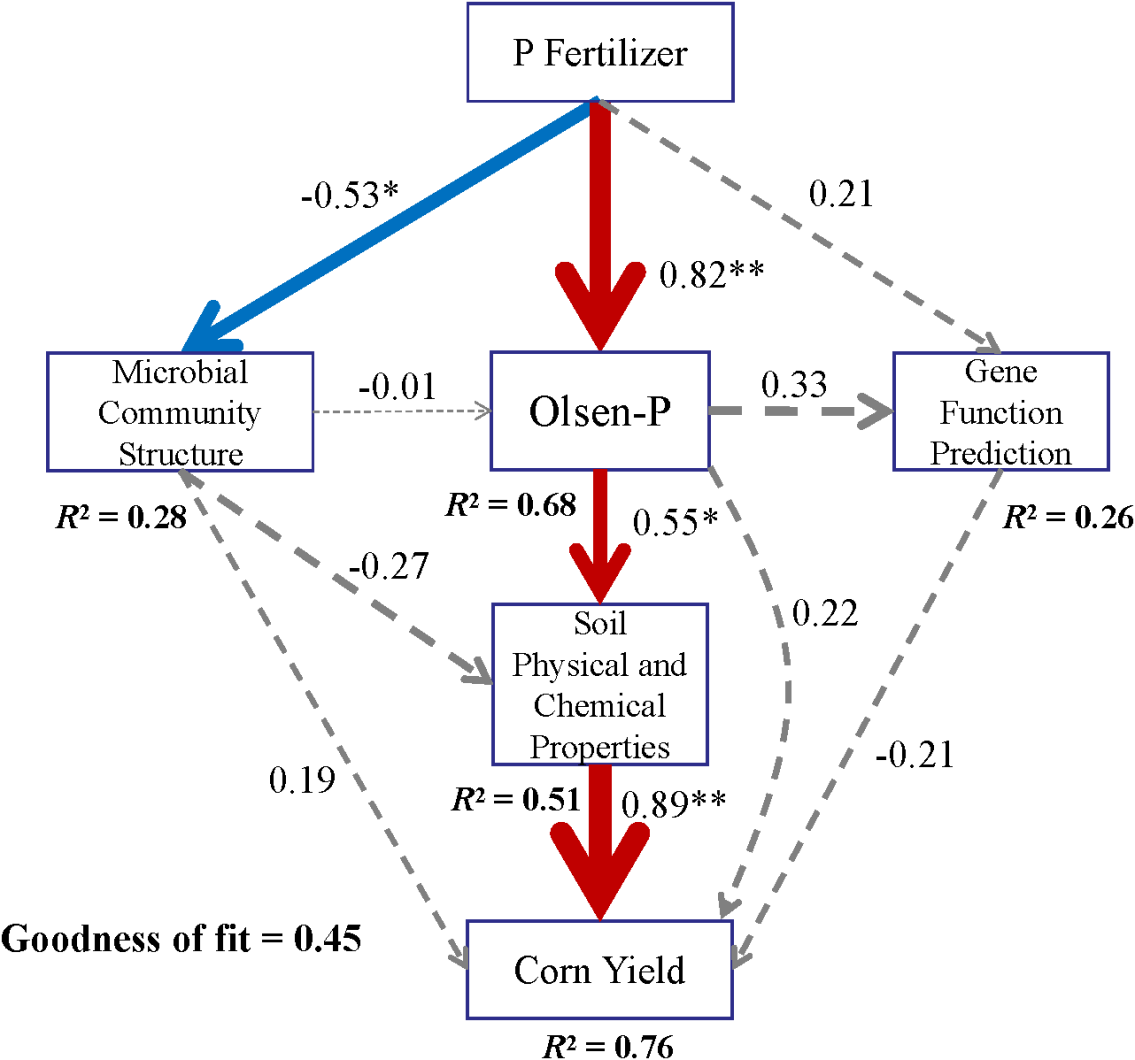
PLS-PM analysis showing the direct and indirect factors affecting maize yield under different P fertilizer applications. *P < 0.05, **P < 0.01.

Partial least-squares path modeling analysis was applied to investigate both direct and indirect relationships among P-fertilizer types, microbial community structure, soil physical properties, AP, functional gene prediction, and maize yield, accounting for 76% of the total variation in maize yield (**Figure 13**). Long-term spatially stratified P fertilization significantly increased maize yield, primarily through its influence on the rhizosphere soil microbial community structure and AP content. Notably, the microbial community structure exerted a positive effect on yield, whereas functional gene prediction showed a non-significant negative association with yield.

## 4 Discussion

### 4.1 Impact of P-fertilizer application on soil microbial community structure and diversity

PCoA revealed significant differences in soil microbial β-diversity, demonstrating that the fungal community structure exhibited relative independence across different P-fertilizer treatments (*P* < 0.05), whereas bacterial communities showed no significant response to fertilizer type. This finding contrasts with several studies reporting significant structural changes in both bacterial and fungal communities under long-term P fertilization in grassland ecosystems (Leff et al., 2015). For example, long-term P-fertilizer application significantly altered the bacterial community structure in wheat field trials conducted in Shaanxi Province, China (Liu et al., 2023a). However, other studies have reported no significant changes in microbial community structure following P-fertilizer application (Hamel et al., 2006; Liu et al., 2012). The absence of significant differences in bacterial community structure may be attributed to either insufficient P supply levels to trigger microenvironmental changes or an inadequate duration of fertilizer application. Furthermore, this study specifically focused on the ultimate impact of long-term stratified fertilization on the microbial community, rather than tracking its temporal dynamics.

Long-term P fertilization exhibited minimal effects on the relative abundance of bacterial and fungal communities at both the phylum and class levels, although the dominant fungal phyla demonstrated greater responsiveness than their bacterial counterparts. This differential sensitivity stems from the inherently higher susceptibility of fungi to soil property alterations and P inputs compared to bacteria (He et al., 2008; Li et al., 2015). Fungal hyphae typically form extensive networks within soil systems, significantly increasing the surface area available for nutrient acquisition and thereby enhancing their capacity to access soil AP compared to other soil microbiota (Smith and Smith, 2011). P-fertilizer application induced significant restructuring of rhizosphere microbial communities (**Figures 5**, **7**), particularly enhancing the complexity and stability of soil fungal assemblages. These modifications would in turn amplify plant–microbe interactions, demonstrating the indispensable role of rhizosphere microbiota in sustaining crop productivity.

### 4.2 Impact of P-fertilizer application on soil functional genes

Investigating the effects of long-term P fertilization on soil functional genes is critical, as C-, N-, and P-cycling genes typically dominate the rhizosphere soil microbiome and play pivotal roles in mediating plant traits and soil properties (Guo et al., 2021). Among agricultural practices, P-fertilizer application has been shown to significantly alter both the abundance and composition of P-cycling functional genes (Lang et al., 2021). Our results demonstrate that spatially stratified MAP application not only significantly increased the relative abundance of plant pathogens within soil fungal functional communities but also enhanced the relative abundance of undefined saprotrophs, indicating the capacity of MAP to improve the fungal community’s competence in litter decomposition and organic matter cycling. Correlation analyses further revealed significant positive relationships of plant pathogens with both AP and TP-R and of undefined saprotrophs with TP-ST. These mechanistic insights explain the superior maize yield enhancement observed under MAP compared to other treatments. Plant pathogens are a primary driver of global agricultural crop losses, as they infect a wide range of host plants and cause severe ecological and economic damage (De Silva et al., 2019; Jayawardena et al., 2021). Integrated management strategies are advocated to mitigate pathogen proliferation, including the application of novel bio-based fungicides (Serrano et al., 2025), crop rotation with non-host species, and optimized irrigation and so on. Notably, fertilization acts as a double-edged sword: while it enhances crop yields, it also elevates soil health risks.

Overall, P-fertilizer application suppressed the abundance of key P-cycling functional genes, including the Pi transporter (*pit*), P transport system permease protein (*phnE*), quinoprotein glucose dehydrogenase (*gcd*), P phosphodiesterase (*phnP*), and triphosphate diphosphatase (*phnM*) genes. This observed downregulation aligns with established patterns of reduced soil P-cycling gene abundance under long-term P supplementation (Bergkemper et al., 2016; Su et al., 2015). Furthermore, a significant positive correlation was observed between *gph* and Acidobacteria abundance (**Figure 12**). MAP exhibited the highest relative abundance of Acidobacteria at 18.38% (**Figure 6**). Notably, *norank_Acidobacteriales*, an Acidobacteria subgroup yet to be classified at the genus level, showed a significant positive correlation with TP-R (**Figure 8**). This is a particularly relevant finding, as the MAP treatment resulted in significantly higher TP-R compared to that under FP (**Figure 2**). These findings collectively suggest that *gph* enhances the root’s P uptake capacity. This interpretation is further supported by the 7.67% higher relative abundance of *gph* in MAP versus other treatments (**Figure 11**), providing mechanistic evidence for MAP’s promotion of root P accumulation through *gph*-mediated processes. *Sphingomonas* (affiliated with the phylum Proteobacteria) exhibited significant negative correlations with soil N, P, AP, TN-S, and TP-S (**Figure 8**). Concurrently, *phoU* showed a significant negative correlation with Proteobacteria (**Figure 12**). MAP demonstrated the lowest Proteobacteria relative abundance (43.20%, **Figure 6**), while exhibiting the highest *phoU* abundance among treatments (**Figure 11**). These findings collectively suggest that *phoU* plays a role in enhancing soil–plant nutrient translocation efficiency. However, the role of this gene in nutrient uptake rate remains unclear, and the specific mechanisms require further investigation. Similarly, *phnA*, a key gene encoding organophosphorus-degrading enzymes, displayed contrasting correlations: a significant positive correlation was found with Proteobacteria but a negative correlation was found with Acidobacteria (**Figure 12**). Notably, MAP treatment showed the lowest *phnA* abundance (3.27%, **Figure 11**), indicating that the nutrient enrichment mechanism of MAP involves suppression of *phnA*-mediated P mineralization pathways.

Nevertheless, the total abundance of bacterial functional genes showed little variation with fertilization regimes, which differs from previous reports indicating that P-fertilizer application reduced the abundance of P-cycling genes involved in PSR regulation (*phoR* and *phoP*) as well as P-uptake and transport (Dai et al., 2019; Hsieh and Wanner, 2010; Ikoyi et al., 2018). This discrepancy may be attributed to the fact that the applied P level in the present experiment was insufficient to significantly alter the relative abundance of functional genes. Alternatively, this discrepancy may arise from the inherent limitations of PICRUSt2 as a predictive tool for microbial metabolic functions, which suggests that metagenomic sequencing techniques are necessary to improve the accuracy and reliability of these findings (Niewiadomska et al., 2020).

### 4.3 The relationship between P-fertilizer application and functional microorganisms

Keystone taxa within microbial communities, irrespective of their relative abundance, play pivotal roles in shaping both community assembly and ecosystem functioning (Banerjee et al., 2018). Notably, *Proteobacteriaand Actinobacteriaare* key players in phosphorus solubilization and mineralization processes, which enhance the decomposition of soil organic matter and substantially contribute to terrestrial phosphorus cycling (Dai et al., 2020; Ragot et al., 2017; Sebastian and Ammerman, 2011). In our study, these two bacterial phyla exhibited significant positive and negative correlations with genes encoding Pi solubilization (*phnA*) and Po mineralization (*phoN, gph, php, ptsP*). Nevertheless, current research on phosphorus-associated fungal functional groups remains limited and warrants further refinement. Collectively, our findings align with previous studies (Fan et al., 2020; Ratliff and Fisk, 2016), confirming that dominant microbial groups in rhizosphere soil are intimately linked to soil functional gene networks.

*Tausonia* exhibited a significant positive correlation with AP. As a saprophytic ascomycete, it promotes plant nutrient uptake by decomposing organic matter (Shi et al., 2024), likely via the secretion of organic acids and phosphatases to solubilize insoluble Pi (e.g., calcium phosphate, iron phosphate) and mineralize Po (Sharma et al., 2013). This process may increase soil AP content and enhance plant P uptake, though this hypothesis remains to be validated and warrants further investigation into the underlying mechanisms. Additionally, STAMP analysis demonstrated that MAP significantly elevated the relative abundance of *Tausonia*. This finding further supports the notion that the MAP treatment’s notable increase in soil AP content underpins its superior performance in yield improvement.

Saprotrophs are organisms that derive energy and nutrients by decomposing dead organic matter via the secretion of extracellular enzymes, playing a pivotal role in nutrient cycling (Gil-Martínez et al., 2018). However, current research on P-associated fungal functional guilds remains limited, with taxonomic frameworks still incomplete. Nevertheless, our findings reveal that P fertilizer application enhances the abundance of Saprotrophic communities in soil, which not only strengthens soil nutrient cycling but also promotes crop root P uptake.

### 4.4 Integrated relationships among maize yield, soil physical properties, AP content, and microbial community structure

Excessive P application disrupts the soil nutrient balance. However, this study demonstrates that stratified fertilization not only helps maintain soil health-conducive microbial communities but also significantly enhances crop yield by increasing the soil AP content. By contrast, previous studies have reported a significant positive correlation between soil functional genes and plant productivity (Fan et al., 2020; Liu et al., 2023a). These discrepancies may stem from variations in cropping systems and soil environmental conditions. Alternatively, this could be attributed to differential mechanisms through which various P fertilizers promote yield increases, consequently leading to distinct effects on grain yield (Guo et al., 2025; Viceli et al., 2025). Furthermore, recent studies indicated that specific microbial taxa interact with soil physical properties and crop yield, which is inconsistent with the findings of this study. This discrepancy may stem from long-term fertilization-induced alterations in soil properties such as pH shifts and organic matter accumulation, which can significantly influence microbial community structure and function (Rousk et al., 2010). In conclusion, long-term reduced P application (commonly used P application rates in Northeast China are 100 kg P_2_O_5_ ha^-1^) can significantly modify the rhizosphere microbial community composition, effectively increase the soil AP content, and improve soil physical properties, ultimately achieving yield enhancement. These improvements are essential for enhancing soil fertility, promoting soil sustainability, and attaining high crop productivity.

### 4.5 Optimizing P-fertilizer management to coordinate soil microbial communities and functions

In our experimental results, the CK soil exhibited the highest bacterial and fungal diversity, while FP significantly reduced the bacterial species richness, indicating that improper P fertilization may disrupt microbial communities and functions (Lang et al., 2021). When comparing the relative abundance of microbial taxa across treatments, we observed that key taxa in the soil are not necessarily correlated with soil health. Although the spatially stratified MAP treatment did not significantly enhance microbial diversity, differential comparisons and correlation analysis revealed that the MAP-treated soil harbored distinct advantageous key taxa and functional compositions (e.g., *Tausonia* and Plant Pathogen). This resulted in a significantly higher Olsen-P content in this treatment compared to that detected for the other fertilizer groups, consequently demonstrating superior performance in increasing maize yield, improving P fertilizer agronomic efficiency, and reducing the soil P surplus relative to other P fertilizers. In contrast, conventional farmer fertilization practices (FP) at equivalent P application rates not only failed to maximize yield increases but also exacerbated P loss and compromised soil health. Previous studies have demonstrated that MAP contributes to improved crop yields. In no-till wheat-soybean cropping systems, the combined application of lime and MAP substantially enhanced crop yields. The AP content varies with different types of P fertilizer, and MAP typically outperforms other treatments in enhancing the soil AP content, which was confirmed in the present study. The MAP-treated soil exhibited an acidic pH (5.66), which is a key factor for enhancing P availability, leading to effective acidification of the rhizosphere environment (Ben Salah et al., 2022). In no-till wheat-soybean cropping systems, the combined application of lime and MAP significantly increased crop yield (Duart et al., 2024). MAP recruits specific microbial taxa to enhance microbial diversity, thereby improving plant biomass through pathways associated with nutrient cycling and energy metabolism. This mechanism further elucidates why MAP exhibits optimal yield-promoting effects under conditions of reduced phosphorus input.

## 5 Conclusion

This study provides the first comprehensive assessment of the systematic effects of long-term stratified P-fertilizer application on soil physical properties, plant nutrient content, microbial community structure, and functional genes in a maize cropping system. Stratified fertilization is an effective strategy for enhancing maize P uptake while minimizing soil P accumulation. Compared with the non-phosphorus treatment, MAP application alleviates fertilizer-induced disruptions to the soil microecological balance. This protective effect is primarily mediated through MAP’s regulation of P-cycling genes, including upregulating *gph* and *phoU* while downregulating *phnA*, which subsequently influences the abundance of dominant taxa such as Acidobacteria and Proteobacteria. These microbial community shifts enhance root nutrient acquisition, concurrently increasing crop yield, reducing the soil P surplus, and balancing P availability. Rational reduction of P input in soils with high Olsen-P levels can align crop P requirements for optimal growth with minimized soil P accumulation and conserved P resources, representing a promising intensive agricultural strategy for maize cropping systems. Furthermore, given the crucial role of soil microbial communities and functional diversity in sustaining maize productivity, greater attention should be directed toward understanding how P fertilizers influence soil microbiota and their ecological functions. These findings offer novel microbial perspectives to advance investigations into the mechanisms of soil–microbe interactions under P fertilization in the black soils of Northeast China, providing feasible approaches to enhance soil P transformation and improve PUE.

## Data Availability

The original contributions presented in the study are publicly available. This data can be found here: the NCBI Sequence Read Archive (SRA) repository (https://www.ncbi.nlm.nih.gov/) under accession numbers SAMN52820566, SAMN52820567, SAMN52820568, SAMN52820569, and SAMN52820570.4.

## Supplementary material

Supplementary Figure 1 Schematic diagram of field experimental regimes.

Figure S2 The average yield of all treatments from 2017 to 2024 (excluding data of 2019).
