# Supplementary figures and images for: Microbial response to long-term spatially stratified phosphorus application in Northeast China
Source: Front Plant Sci. 2025 Oct 23;16:1669876. doi: 10.3389/fpls.2025.1669876 (PMC12589036; doi:10.3389/fpls.2025.1669876)

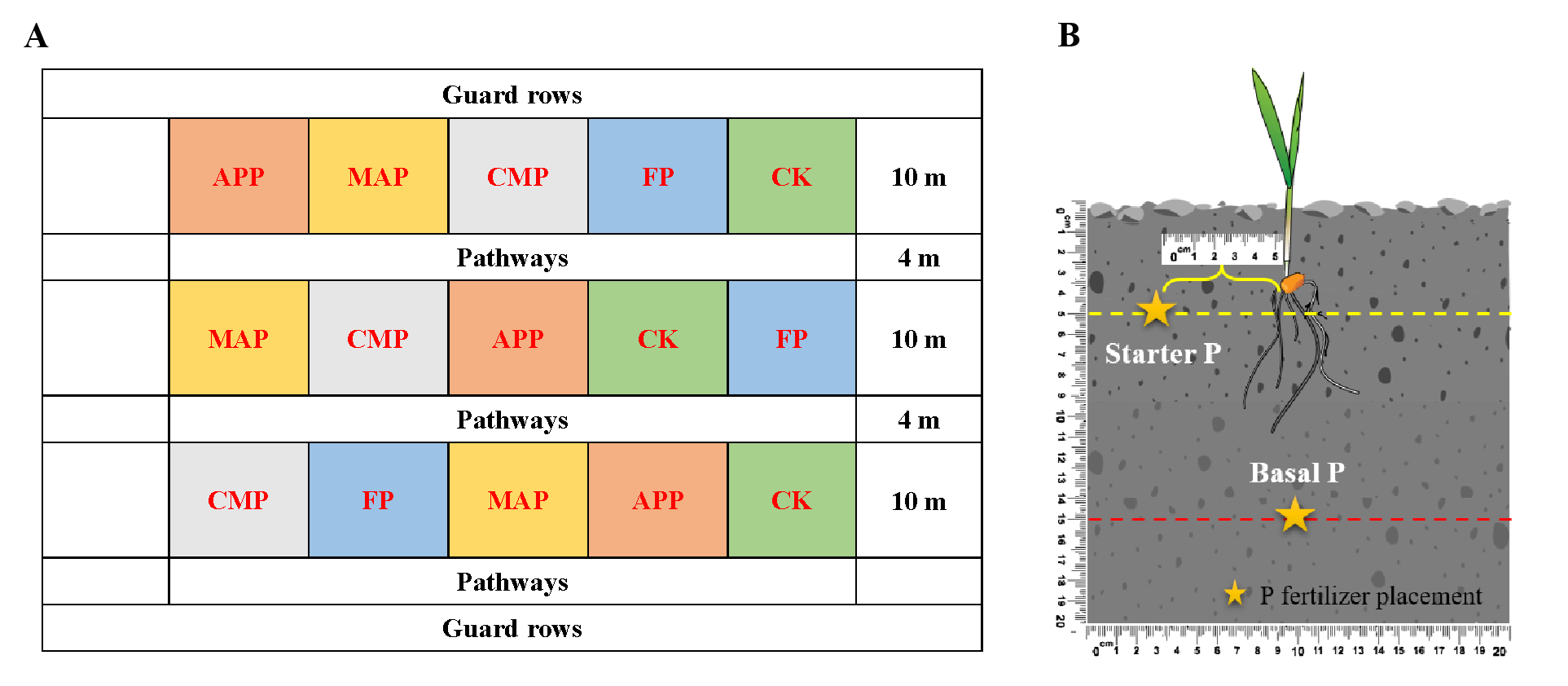

Supplement: Supplementary Figure 1 — Schematic diagram of field experimental regimes. [file Image1.tif]

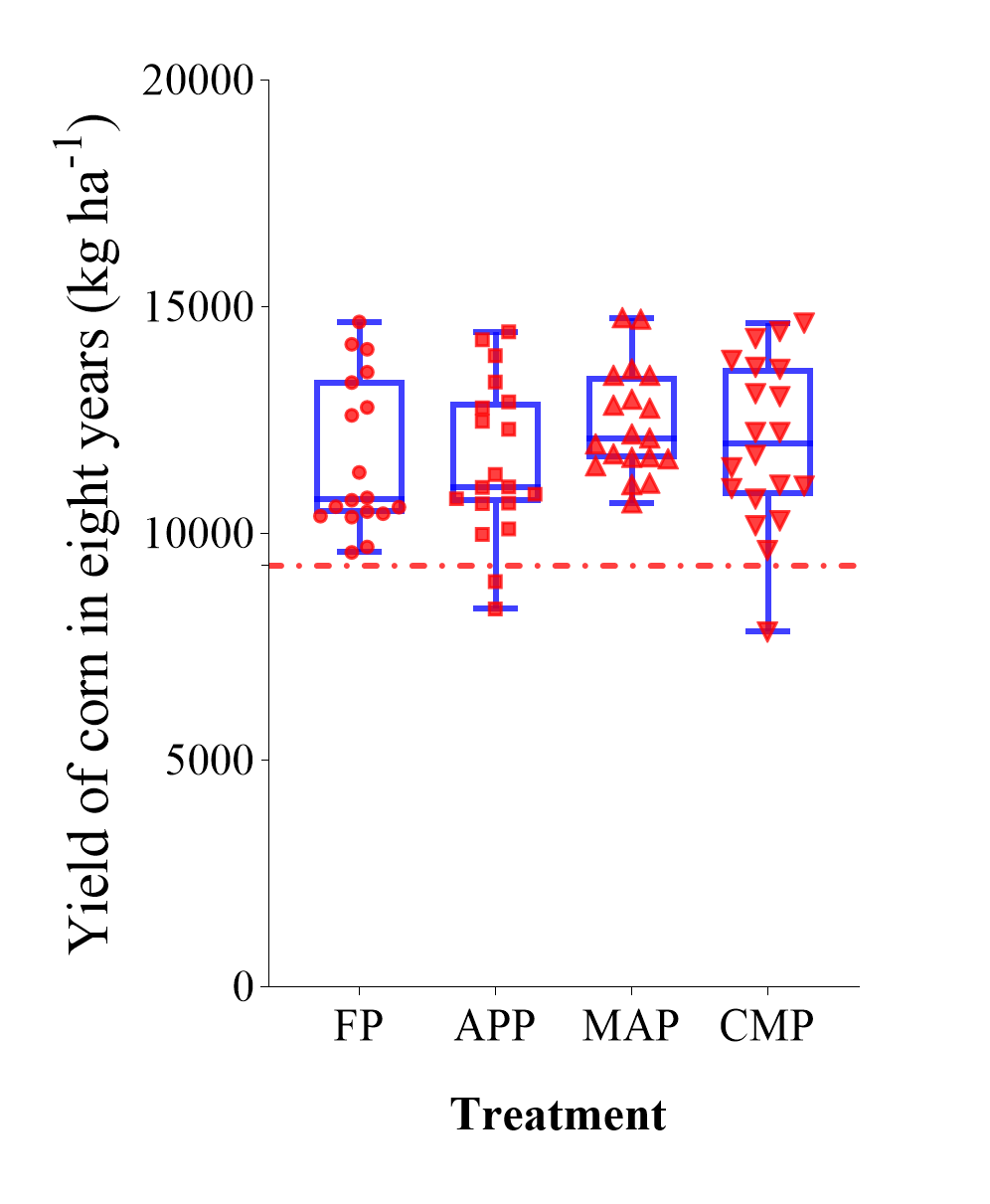

Supplement: Figure S2 — The average yield of all treatments from 2017 to 2024 (excluding data of 2019). [file Image2.tif]
