# Supplementary material for: Microbial response to long-term spatially stratified phosphorus application in Northeast China
Source: Front Plant Sci. 2025 Oct 23;16:1669876. doi: 10.3389/fpls.2025.1669876 (PMC12589036; doi:10.3389/fpls.2025.1669876)
Supplement: Supplementary file 3 [file Table1.docx]

Microbial Response to Long-term Spatially Stratified Phosphorus Application in Northeast China

Liyuan Hou^†^, Bing Han^†^, Yixin Wang, Xiaoli Wang, Wuliang Shi, Ning Cao^*^, Yubin Zhang^*^

College of Plant Science, Jilin University, Changchun, China

**†These authors have contributed equally to this work and share the first authorship.**

*** Correspondence:**Corresponding Author
Ning Cao, [cao_ning@jlu.edu.cn](mailto:cao_ning@jlu.edu.cn); Yubin Zhang, [ybzhang@jlu.edu.cn](mailto:ybzhang@jlu.edu.cn)

Keywords: spatially stratified fertilization, phosphorus efficiency, maize yield, microbial community structure, functional genes

Table S1 Types and methods of fertilizer application in field experiments

| **Types and application rates of fertilizers（kg ha^-1^ P_2_O_5_)** | | | | | |
| --- | --- | --- | --- | --- | --- |
| **The treatments** | **Base fertilizer** | **Starter fertilizer** | | | **Note** |
|  |  | **APP** | **MAP** | **CMP** |  |
| CK | 0 |  |  |  | Control group |
| FP | 75 |  |  |  | Farmer's Practice |
| APP | 60 | 15 |  |  |  |
| MAP | 60 |  | 15 |  |  |
| CMP | 60 |  |  | 15 |  |

Table S2 The KO number, function descriptions, gene name and classification of the investigated genes referring to KEGG database.

| **Classification** | **KO number** | **Details for gene function** | **Corresponding gene** | |
| --- | --- | --- | --- | --- |
| Genes involved in  P-starvation response regulation | K02039 | phosphate transport system protein | | *phoU* |
|  | K07636 | two-component system, OmpR family, phosphate regulon sensor histidine kinase PhoR | | *phoR* |
|  | K07657 | two-component system, OmpR family, phosphate regulon response regulator PhoB | | *phoB* |
|  | K07658 | two-component system, OmpR family, alkaline phosphatase synthesis response regulator PhoP | | *phoB1* |
| Genes involved in P-uptake and transport system | K05813 | sn-glycerol 3-phosphate transport system substrate-binding protein | | *ugpB* |
|  | K05814 | sn-glycerol 3-phosphate transport system permease protein | | *ugpA* |
|  | K05815 | sn-glycerol 3-phosphate transport system permease protein | | *ugpE* |
|  | K05816 | sn-glycerol 3-phosphate transport system ATP-binding protein | | *ugpC* |
|  | K02041 | phosphonate transport system ATP-binding protein | | *phnC* |
|  | K02042 | phosphonate transport system permease protein | | *phnE* |
|  | K02044 | phosphonate transport system substrate-binding protein | | *phnD* |
|  | K02036 | phosphate transport system ATP-binding protein | | *pstB* |
|  | K02037 | phosphate transport system permease protein | | *pstC* |
|  | K02038 | phosphate transport system permease protein | | *pstA* |
|  | K02040 | phosphate transport system substrate-binding protein | | *pstS* |
|  | K03306 | inorganic phosphate transporter, PiT family | | *pit* |
| Inorganic P-solublization | K00117 | quinoprotein glucose dehydrogenase | | *gcd* |
|  | K01507 | inorganic pyrophosphatase | | *ppa* |
|  | K01524 | exopolyphosphatase / guanosine-5'-triphosphate,3'-diphosphate pyrophosphatase | | *ppx* |
|  | K06193 | protein PhnA | | *phnA* |
|  | K06136 | pyrroloquinoline quinone biosynthesis protein B | *pqqB* | |
|  | K06137 | pyrroloquinoline-quinone synthase | *pqqC* | |
|  | K06138 | pyrroloquinoline quinone biosynthesis protein D | *pqqD* | |
|  | K06139 | pyrroloquinoline quinone biosynthesis protein E | *pqqE* | |
|  | K00112 | glycerol-3-phosphate dehydrogenase subunit B | *glpB* | |
|  | K00113 | glycerol-3-phosphate dehydrogenase subunit C | *glpC* | |
| Organic P-mineralization | K05774 | ribose 1,5-bisphosphokinase | *phnN* | |
|  | K05780 | alpha-D-ribose 1-methylphosphonate 5-triphosphate synthase subunit PhnL | *phnL* | |
|  | K05781 | putative phosphonate transport system ATP-binding protein | *phnK* | |
|  | K06162 | alpha-D-ribose 1-methylphosphonate 5-triphosphate diphosphatase | *phnM* | |
|  | K06163 | alpha-D-ribose 1-methylphosphonate 5-phosphate C-P lyase | *phnJ* | |
|  | K06164 | alpha-D-ribose 1-methylphosphonate 5-triphosphate synthase subunit PhnI | *phnI* | |
|  | K06165 | alpha-D-ribose 1-methylphosphonate 5-triphosphate synthase subunit PhnH | *phnH* | |
|  | K06166 | alpha-D-ribose 1-methylphosphonate 5-triphosphate synthase subunit PhnG | *phnG* | |
|  | K06167 | phosphoribosyl 1,2-cyclic phosphate phosphodiesterase | *phnP* | |
|  | K02043 | GntR family transcriptional regulator, phosphonate transport system regulatory protein | *phnF* | |
|  | K05306 | phosphonoacetaldehyde hydrolase | *phnX* | |
|  | K03430 | 2-aminoethylphosphonate-pyruvate transaminase | *phnW* | |
|  | K01093 | 4-phytase / acid phosphatase | *appA* | |
|  | K07048 | phosphotriesterase-related protein | *php* | |
|  | K01126 | glycerophosphoryl diester phosphodiesterase | *ugpQ* | |
|  | K01077 | alkaline phosphatase | *phoA* | |
|  | K01113 | alkaline phosphatase D | *phoD* | |
|  | K09474 | acid phosphatase (class A) | *phoN* | |
|  | K03788 | acid phosphatase (class B) | *aphA* | |
|  | K01078 | acid phosphatase | *PHO* | |
|  | K01079 | phosphoserine phosphatase | *serB* | |
|  | K01091 | phosphoglycolate phosphatase | *gph* | |
|  | K08483 | phosphotransferase system, enzyme I, PtsI | *ptsI* | |
|  | K08484 | phosphotransferase system, enzyme I, PtsP | *ptsP* | |
